# Crystal structures of two new high-pressure oxynitrides with composition SnGe_4_N_4_O_4_, from single-crystal electron diffraction

**DOI:** 10.1107/S2052520624002683

**Published:** 2024-05-08

**Authors:** Philipp Gollé-Leidreiter, Shrikant Bhat, Leonore Wiehl, Qingbo Wen, Peter Kroll, Ryo Ishikawa, Martin Etter, Robert Farla, Yuichi Ikuhara, Ralf Riedel, Ute Kolb

**Affiliations:** a Glass and Mineral Materials, Fraunhofer ISC, Neunerplatz 2, Würzburg, 97082, Germany; b Institute for Applied Geosciences, Technische Universität Darmstadt, Schnittspahnstraße 9, Darmstadt, 64287, Germany; c Deutsches Elektronen-Synchrotron, DESY, Notkestr. 85, Hamburg, 22607, Germany; d FB Materialwissenschaft / FG Disperse Feststoffe, Technische Universität Darmstadt, Otto Berndt-Str. 3, Darmstadt, D-64287, Germany; e State Key Laboratory of Powder Metallurgy, Central South University, Changsha, 410083, People’s Republic of China; f Department of Chemistry and Biochemistry, University of Texas at Arlington, Arlington, Texas 76019, USA; g Institute of Engineering Innovation, University of Tokyo, 2-11-16 Yayoi, Bunkyo-ku, Tokyo, 113-8656, Japan; h Institute for Physical Chemistry, Johannes Gutenberg-Universität, Welderweg 11, Mainz, 55099, Germany; University of Antwerp, Belgium

**Keywords:** 3D electron diffraction, high pressure–high temperature (HP-HT) synthesis, oxynitrides

## Abstract

SnGe_4_N_4_O_4_ was synthesized at high pressure (16 and 20 GPa) and high temperature (1200 and 1500°C) in a large-volume press. Powder X-ray diffraction experiments using synchrotron radiation indicate that the derived samples are mixtures of known and unknown phases. However, the powder X-ray diffraction patterns are not sufficient for structural characterization. Transmission electron microscopy studies reveal crystals of several hundreds of nanometres in size with different chemical composition. Among them, crystals of a previously unknown phase with stoichiometry SnGe_4_N_4_O_4_ were detected and investigated using automated diffraction tomography (ADT), a three-dimensional electron diffraction method. Via ADT, the crystal structure could be determined from single nanocrystals in space group *P*6_3_ *mc*, exhibiting a nolanite-type structure. This was confirmed by density functional theory calculations and atomic resolution scanning transmission electron microscopy images. In one of the syntheses runs a rhombohedral 6*R* polytype of SnGe_4_N_4_O_4_ could be found together with the nolanite-type SnGe_4_N_4_O_4_. The structure of this polymorph was solved as well using ADT.

## Abstract 2

A new ternary oxynitride SnGe_4_N_4_O_4_ has been synthesized under high-pressure high-temperature conditions in a large-volume press. Its crystal structure (*P*6_3_ *mc*) and the crystal structure of a 6*R* polytype (*R* 3 *m*) were determined by three-dimensional electron diffraction from submicrometre-sized crystals.

## 1. Introduction

Germanium and tin are Group 14 elements, capable of forming nitrides and oxynitrides (Bhat *et al.*, 2020 ▸; Jorgensen *et al.*, 1979 ▸; Labbe & Billy, 1977 ▸) with remarkable thermomechanical and optoelectronic properties. Spinel-type germanium and tin nitrides were synthesized long ago (Serghiou *et al.*, 1999 ▸; Scotti *et al.*, 1999 ▸). Their solid solutions are predicted to form wide direct bandgap semiconductors (Boyko *et al.*, 2013 ▸, 2010 ▸). Sinoite-type germanium oxynitride Ge_2_N_2_O has been reported with orthorhombic symmetry (Labbe & Billy, 1977 ▸). Tin oxynitride Sn_2_N_2_O has been found to form a Rh_2_S_3_-type structure in space group *Pbcn*, synthesized at high-pressure high-temperature (HP-HT) conditions (Bhat *et al.*, 2020 ▸).

GeO_2_ and SnO_2_ crystallize in the rutile structure type (Haines & Léger, 1997 ▸; Shiraki *et al.*, 2003 ▸) at ambient conditions and show the same structure types under high pressure. No solid solutions of these compounds are known. Only a limited maximum solubility of 4 mol% GeO_2_ in rutile-type SnO_2_ at 1250°C has been reported (Watanabe *et al.*, 1983 ▸). Mixed GeSn nitrides or oxynitrides are unreactive at ambient conditions and have not been reported up to now, nor are there any computational studies on mixed GeSn oxynitride compounds to the best of our knowledge. This can be explained by Goldschmidt’s ninth rule, which postulates that isomorphic substitution between different ions is only possible if the difference in ionic radii is less than 15% of the smaller ion (Goldschmidt, 1926 ▸). For Ge and Sn this difference is 30% [taking the ionic radii for octahedral coordination from Shannon, (1976 ▸)].

In the case of alloys, bulk Ge–Sn solutions with diamond structure were synthesized at HP-HT conditions by Serghiou *et al.* (2021 ▸). This particularly highlights the high-pressure requirement for the compatibility of Ge and Sn.

Here we report for the first time a GeSn oxynitride, SnGe_4_N_4_O_4_, with a nolanite-type crystal structure and a rhombohedral polytype. Nolanite is an oxide mineral with a hexagonal crystal structure, space group *P*6_3_ *mc* and composition (V, Fe, Ti, Al)_5_O_7_OH (Gatehouse *et al.*, 1983 ▸; Hanson, 1958 ▸). Other minerals with a nolanite-type structure have also been described, *e.g.* akdalaite (= thodite) Al_5_O_7_OH (Yamaguchi *et al.*, 1969 ▸) or rinmanite (Sb, Zn, Mn, Mg)_2_(Fe,Mg)_3_O_7_OH (Holtstam *et al.*, 2001 ▸). The crystal chemistry of nolanite-type molybdates *A*_2_Mo_3_O_8_ has been investigated in detail for a wide range of metals, with *A* = Mg, Mn, Fe, Co, Ni, Zn, Cd by McCarroll *et al.* (1957 ▸) and *A* = Mg, Co, Zn, Mn by Abe *et al.* (2010 ▸). Hitherto, no nitrides or oxynitrides with nolanite-type structure were reported. In this work, we focus on the novel phase SnGe_4_N_4_O_4_, which was detected in three different HP-HT experiments.

## 2. Experimental

### 2.1. Synthesis

HP-HT synthesis experiments were performed using ‘Aster-15’, the large-volume press installed at the P61B beamline of PETRA III (Farla *et al.*, 2022 ▸) at DESY, Hamburg. All HP-HT experiments were carried out using the 14 mm or 10 mm MgO (Cr_2_O_3_-doped) octahedron and 7 mm or 4 mm truncated tungsten carbide (Fujilloy, 32 mm) anvils. Lanthanum chromite was used as a resistive heater material. Actual pressure and temperature exerted on the sample were calculated from the calibrated relationships between oil pressure (bar) and pressure (GPa), and between heating power (W) and temperature (°C). The starting materials were amorphous oxygen-containing Sn–Ge–N precursors, which had been prepared by the polymer-derived ceramic route (Riedel, 2023 ▸), inspired by previously established reactions between Sn(NEt_2_)_4_ or Hf(NEtMe)_4_ and condensed NH_3_ (Li *et al.*, 2016 ▸; Salamat *et al.*, 2013 ▸) (Fig. S1). The HP-HT conditions were ∼20 GPa, ∼1500°C (run #HH228, Pt capsule) and ∼16 GPa, ∼1200°C (run #HH266, graphite capsule). In contrast to sample #HH228, the transfer of the precursor material to the HP capsule was performed for sample #HH266 in a glovebox under an inert gas atmosphere to prevent oxygen contamination. In both experiments, a mixture of several different crystalline phases was formed. One of these phases, common to both samples, is the novel oxynitride SnGe_4_N_4_O_4_ described in this work. To confirm the formation and reproducibility of the novel oxynitride phase, a follow-up HP-HT synthesis experiment (run #HH670, 15.6 GPa, ∼1200°C, graphite capsule) was conducted, using a mechanical mixture of Ge_2_N_2_O and SnO_2_ (2:1 molar ratio) as the starting material.

### 2.2. Synchrotron powder X-ray diffraction

A qualitative phase analysis, including the refinement of the unit-cell parameters, was performed by angle-dispersive powder X-ray diffraction, using synchrotron radiation at the powder diffraction and total scattering beamline P02.1 of PETRA III at DESY (Hamburg, Germany). P02.1 operates at a fixed energy of 60 keV (∼0.207 Å). The samples were recovered chunks from the HP-HT runs. They were mounted in tight-fitting kapton capillaries with diameters of 0.8 mm to 1.2 mm. The samples were always spun during measurement to avoid any texture effect. The diffraction patterns with an angular range of 1° to 16° were recorded in transmission geometry on an area detector.

### 2.3. TEM, ADT and TEM-EDX

Three-dimensional electron diffraction (3D ED) and energy-dispersive X-ray spectroscopy (EDX) measurements were conducted using a Tecnai F30 ST instrument with an acceleration voltage of 300 kV (wavelength 0.0197 Å) at the University of Mainz. For 3D ED the illumination system was set to μ-STEM mode with a beam diameter of 200 nm (gun lens 8, spot size 6, C2 condenser aperture 10 µm). The high-angle annular dark-field (HAADF) images for crystal tracking were measured with a Fischione detector. The diffraction patterns were acquired in nanobeam electron diffraction (NBED) mode, with additional electron beam precession (semi-angle of 1° and frequency of 100 Hz (Nanomegas Digistar). A single tilt holder with a tilt range of ±70° from Fischione allowed for the acquisition of 3D ED data on a Gatan US4000 4k × 4k 16-bit CCD camera. To control the 3D ED measurement the Fast-ADT module (Plana-Ruiz *et al.*, 2020 ▸) was used in sequential mode. An EDAX Si(Li) detector was used to acquire the EDX spectra at a stage tilt of 20°. The EDX spectra were quantified using the Emispec *ESVision* software.

TEM samples were prepared by crushing the material in an agate mortar, suspending the resulting powder in ethanol and dropping the suspension on copper grids coated with a continuous carbon film.

Reconstruction of the reciprocal space was performed with *eADT* (Kolb *et al.*, 2019 ▸). The reflection files were prepared with *PETS2.0* (Palatinus *et al.*, 2019 ▸). Crystal structure determination was carried out with *SIR*2014 (Burla *et al.*, 2015 ▸) at 0.8 Å resolution and refinement of the crystal structure was carried out with *JANA2020* (Petříček *et al.*, 2014 ▸). All reflections with *I* < 3σ were classified as unobserved. The structure model was first refined using kinematical approximation. The resulting structure model was further refined, taking dynamical scattering into account (Palatinus *et al.*, 2015 ▸).

### 2.4. Density functional theory

Density functional theory (DFT) calculations were performed with the Vienna *ab initio* simulation package (VASP) (Hohenberg & Kohn, 1964 ▸; Kresse & Furthmüller, 1996 ▸; Kresse & Hafner, 1993 ▸, 1994 ▸) using the strongly conserved and appropriately normed (SCAN) functional together with the projector-augmented-wave (PAW) method (Blöchl, 1994 ▸; Kresse & Joubert, 1999 ▸; Sun *et al.*, 2015 ▸). Reported results refer to calculations with a plane wave cut-off energy of 500 eV. The Brillouin zone of the nolanite-type unit cell and all derived structures was sampled using a 6 × 6 × 4 **k**-point mesh. With the parameters reported above, enthalpy differences between structures are converged to better than 0.01 eV per conventional unit cell and less than 1 meV per atom.

### 2.5. Atomic resolution scanning transmission electron microscopy

The electron transparent thin TEM sample was prepared by a focused ion beam, using a Helios G5 focused ion beam scanning electron microscope (FIB-SEM) (Thermo Fisher Scientific), at the University of Tokyo. Atomic resolution annular dark-field (ADF) and annular bright-field (ABF) scanning transmission electron microscopy (STEM) images were acquired with a JEOL ARM300CF transmission electron microscope installed at the University of Tokyo. The ARM300CF is equipped with a Delta-type corrector and a cold field-emission gun and was operated with an acceleration voltage of 300 kV. The illumination semi-angle used was 20 mrad, and the collection semi-angles of ADF and ABF detectors were 40–200 mrad and 10–20 mrad, respectively. To suppress the electron beam damage of the sample, a considerably low electron beam current of 3 pA was used. To enhance the signal-to-noise ratio of the images, sequential fast-scanning STEM imaging was performed and 10 frames were averaged after the image alignment with the rigid-body translation algorithm (Ishikawa *et al.*, 2014 ▸).

## 3. Results

Both recovered samples #HH228 and #HH266 were investigated using synchrotron powder X-ray diffraction (PXRD). Depending on the applied HP-HT synthesis conditions, they show mixtures of several crystalline phases. Among them, only one known phase was identified, namely an α-PbO_2_-type high-pressure polymorph of SnO_2_ (Haines & Léger, 1997 ▸) in sample #HH228. Most of the reflections could not be ascribed to any known Sn or Ge-containing phase. Details of the further analysis are given in Section 3.4.

### 3.1. SEM

**Figure 1:**
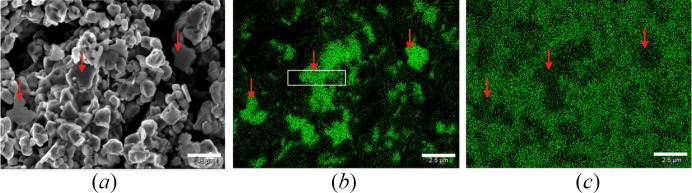
Simultaneously acquired: (*a*) BSE SEM image of the recovered sample #HH266, and EDX mapping of (*b*) Ge-*L* edge and (*c*) Sn-*L* edge. White scale bar = 2.5 µm. Red arrows indicate Ge-enriched grains. The white rectangle indicates the position of FIB sampling.

According to the synchrotron XRD analysis, sample #HH266 contains a mixture of different phases. To confirm the distribution of these phases, backscattered electron (BSE) imaging and energy-dispersive X-ray spectroscopy (EDX) was performed. Fig. 1 ▸(*a*) shows the BSE SEM image of the surface of sample #HH266. The grain sizes are estimated to be in the range of 0.2 to 2 µm. Oxygen and nitro­gen atoms are uniformly distributed throughout the whole sample. Nevertheless, different Sn to Ge ratios were found for different grains, indicated by red arrows in Fig. 1 ▸(*b*). As indicated by red arrows in Fig. 1 ▸, the Ge-enriched grains have a platy morphology, which may hint at a unique symmetry axis. To directly image the atomic structure of the germanium-enriched grains, the grain marked by the white rectangle in Fig. 1 ▸(*b*) was prepared by FIB milling and observed by atomic resolution STEM imaging. This will be discussed in Section 3.7.

### 3.2. TEM-EDX

**Figure 2:**
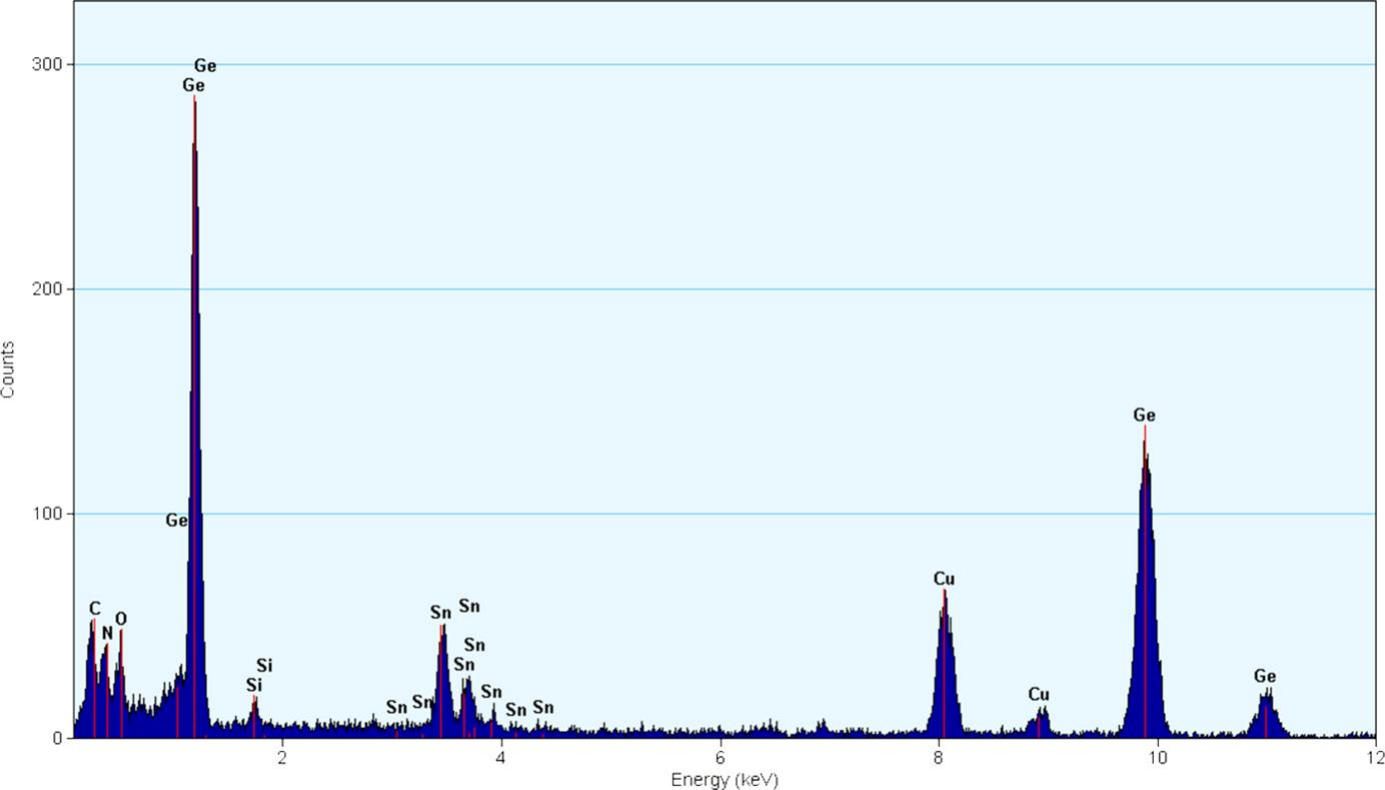
EDX spectrum up to 12 kV of the novel Ge-rich phase from #HH228. C, Cu and Si peaks originate from the TEM grid and the Si(Li)-EDX detector, respectively.

Both HP-HT products were well crystallized, with a grain size of several hundred nanometres. The SnGe_4_N_4_O_4_ phase, which is the focus of this work, could be distinguished from other phases by EDX measurements in both the TEM and SEM. In both HP-HT samples, single crystals of a phase with a very prominent Ge peak and a much smaller additional Sn peak could be identified. More detailed EDX spectra of the single crystals were taken with higher dose to confirm the presence of both N and O, indicating that the crystals are oxynitrides. A typical EDX spectrum is shown in Fig. 2 ▸ (additional spectra are shown in Figs. S5 and S6).

The quantification of these spectra indicates a ratio of Sn to Ge of around 0.25 for the single nolanite-type crystals from #HH228 and 0.18 for the corresponding crystals from #HH266. The height of the O and N signal suggests an approximate 1:1 ratio of O to N, but due to the close proximity of the C, N and O ionization edges a reliable quantification is not possible (Tessier, 2018 ▸).

### 3.3. ADT

The crystals in both samples are too small for single-crystal structure determination by X-ray diffraction, therefore 3D ED was used instead. The advantage of 3D ED is that a crystal structure determination can be performed on a single nanocrystal (Kolb *et al.*, 2007 ▸; Gemmi *et al.*, 2019 ▸). Thus, even nanocrystalline mixtures of different phases can be investigated. 3D ED has been previously used to solve structures from materials synthesized in large-volume press experiments (Gemmi *et al.*, 2011 ▸; Bhat *et al.*, 2015 ▸, 2020 ▸).

Four ADT datasets were analyzed in this work and labeled Cr1 to Cr4. Cr1 originates from sample #HH228 and Cr2, Cr3 and Cr4 from sample #HH266. Cr1_HH228 and Cr2_HH266 are datasets of the nolanite-type phase, while Cr3_HH266 and Cr4_HH266 are datasets of the 6*R* polytype. Unit-cell parameters of the crystal structures derived using ADT were refined subsequently from synchrotron PXRD data. Results are provided in Section 3.4.

#### 3.3.1. Sample #HH228

ADT measurements, using a tilt range of −70° to 60°, were performed on SnGe_4_N_4_O_4_ crystals identified via EDX. After data processing a hexagonal unit cell with parameters around *a* = 5.88 Å, *b* = 5.84 Å, *c* = 9.40 Å could be found from ADT measurements.

**Figure 3:**
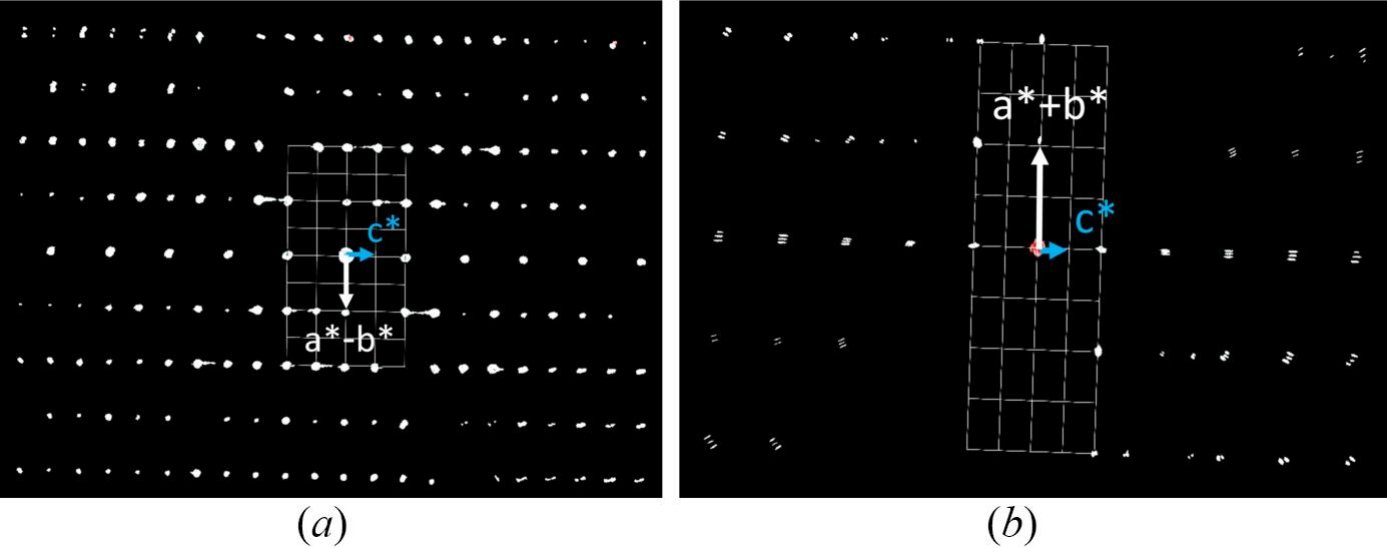
Slices of the reconstructed reciprocal space of the SnGe_4_N_4_O_4_ phase of Cr1_HH228. (*a*) Viewing direction [110] showing the 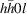 reflections. For 000*l* reflections, *l* = 2*n* is clearly visible, no other extinctions are visible. (*b*) Viewing direction 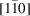 showing the 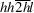 reflections, *l* = 2*n* is only violated by weak reflections. The grid shown in the figures represents the corresponding projections of the reciprocal unit cell onto the image plane. As a* and b* are not parallel to this plane in the given viewing directions, the grid points representing the projection of a* or b* onto the image plane are not occupied by reflections in the zero layer of the reciprocal space, however in higher layers above and below.

For this unit cell, systematic extinctions could be found along the *c*-axis for 000*l* with only reflections *l* = 2*n* being present, corresponding to a 2_1_ or 6_3_ screw axis [Fig. 3 ▸(*a*)], and for the reflection condition 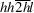 with *l* = 2*n*, giving a *c*-glide plane perpendicular to the 〈210〉 directions [Fig. 3 ▸(*b*)]. As no global extinctions were observed, this results in an extinction symbol of *P* – – *c* (trigonal/hexagonal) (Fig. 3 ▸). A close inspection of the 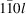 and 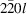 reflections shows slight diffuse scattering in the c* direction.

The crystal structure of SnGe_4_N_4_O_4_ could be solved in space groups *P*31*c* and *P*6_3_ *mc* from the dataset Cr1_HH228. The higher symmetry space group *P*6_3_ *mc* was chosen because the structure solution and refinement showed no features which would justify the introduction of the additional free parameters of the lower-symmetry space group *P*31*c*. Two other hexagonal space groups, namely *P*6_3_/*mmc* and 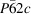 , are also compatible with the observed extinctions. However, these space groups do not fit to the structure solution, because both contain an additional mirror plane perpendicular to the *c*-axis. The crystal structure was refined to an *R*(obs) value of 19.41% using kinematic approximation (completeness 86.6%, resolution 0.5 Å, independent reflections 788). With consideration of dynamical effects, *R*(obs) decreased to 9.42%. The resulting atomic positions and equivalent isotropic displacement parameters are listed in Table 1 ▸ and the crystal structure is shown in Section 3.5.

#### 3.3.2. Sample #HH266

In sample #HH266 the same nolanite-type phase as in #HH228 was found, although with slightly shorter *a* and *b* axes. ADT data, with tilt range ±60°, delivered unit-cell parameters of *a* = 5.82 Å, *b* = 5.84 Å and *c* = 9.40 Å.

**Figure 4:**
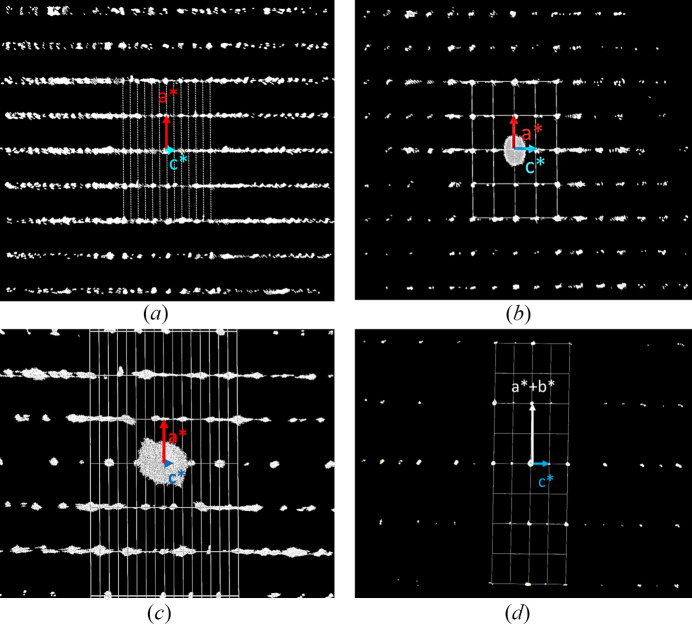
Images of the reconstructed reciprocal space. (*a*) The projection of the reciprocal space of Cr3_HH266 viewed down [010] showing the unit cell of the 6*R* polytype with the threefold superstructure along c*. (*b*) The [010] projection of Cr2_HH266 with much less additional reflections along c* compared to (*a*). The reciprocal unit cell corresponds to the nolanite-type phase. (*c*) 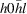 reflections of Cr3_HH266, *i.e.* a single slice of the reciprocal space, cut out of the projection in (*a*). In comparison to Cr2_HH266 additional streaks are visible. (*d*) 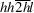 reflections of Cr2_HH266. The systematic extinctions of the *c*-glide plane are slightly violated due to additional reflections from the rhombohedral phase. The grid represents the projection of the reciprocal unit cell onto the image plane.

Moreover, the diffraction patterns of most crystals from sample #HH266 display streaks along the *c*-axis, as well as additional reflections from a second phase [Fig. 4 ▸(*c*)]. This phase has the extinction symbol *R* – – with *a* ≃ 5.8 Å (equal to the nolanite-type phase) and a tripled *c*-axis of around 28.2 Å [Fig. 4 ▸(*a*)], hiding some of the systematic extinctions of the nolanite-type phase [Fig. 4 ▸(*d*)].

**Figure 5:**
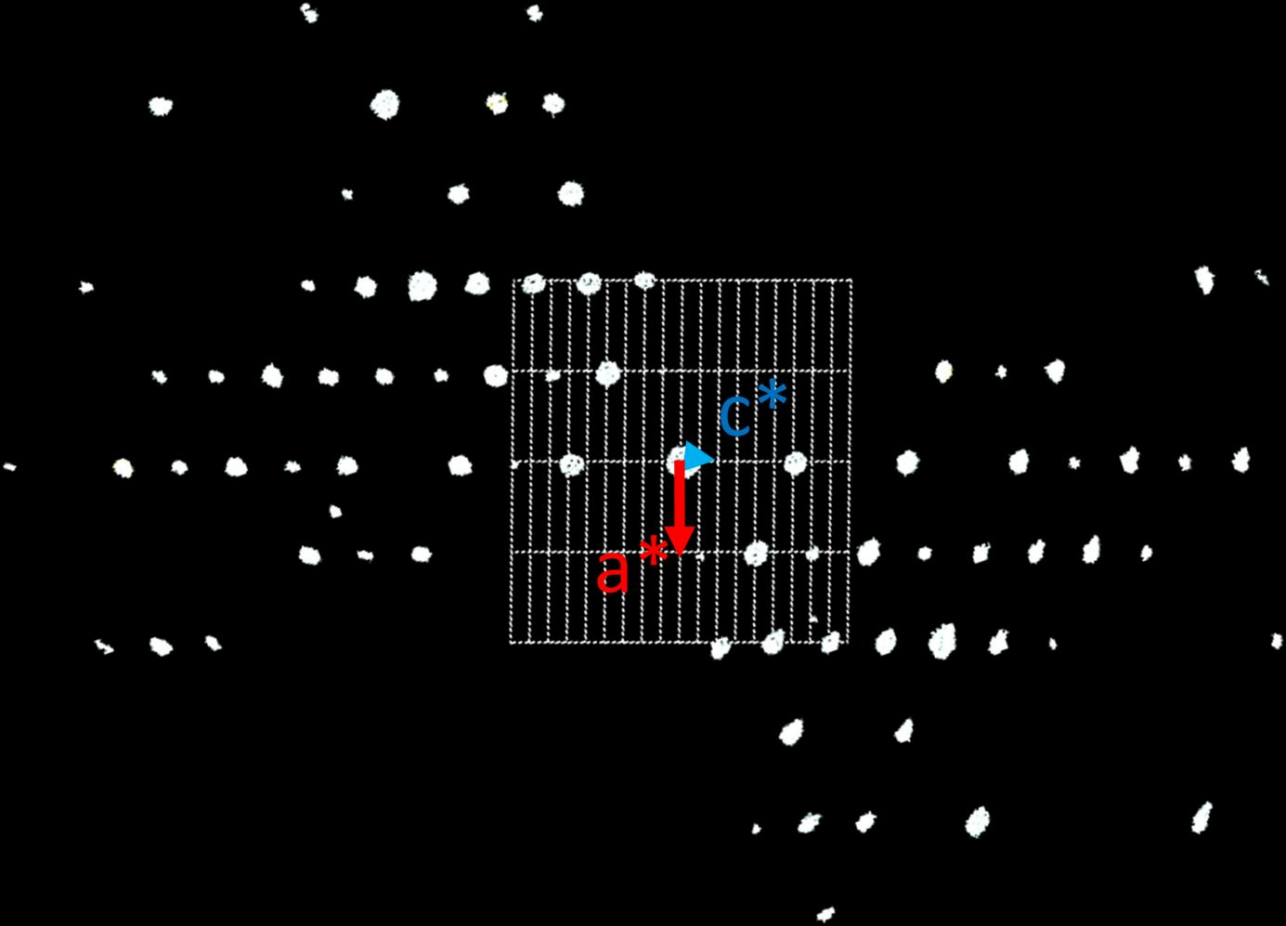
Reciprocal space of Cr4_HH266. Slice of the 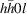 reflections, showing the presence of an obverse rhombohedral centering with *hkil*: −*h*+*k*+*l* = 3*n*. The *l* = 2*n* condition for 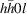 reflections indicating a *c*-glide plane is violated giving *R* – – as an extinction symbol.

Most of the measured crystals in sample #HH266 exhibit reflections of both the nolanite-type and the 6*R* polytype with varying relative amounts. Crystal Cr2_HH266 shows very strong intensities of the nolanite-type phase, while for Cr3_HH266 the reflections of the rhombohedral phase are much stronger. Only crystal Cr4_HH266 provided exclusively the reflections of the rhombohedral phase (Fig. 5 ▸). It did not show any streaks, which suggests that the observed streaks in the other crystals are due to the two phases exhibiting stacking faults at their phase boundaries.

Structure solution and refinement of the nolanite-type phase were possible from the dataset Cr2_HH266 (completeness 94.9%, resolution 0.5 Å, independent reflections 465), which shows only weak additional reflections of the rhombohedral phase. The crystal structure could be refined kinematically to an *R*(obs) value of 21.28% and dynamically to an *R*(obs) value of 11.25% (Table 2 ▸).

The crystal structure of the second phase could be determined in space group 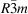 from datasets Cr4_HH266 (tilt range −40° to 30°, completeness 96.6%, resolution 0.63 Å, independent reflections 502) and Cr3_HH266 (tilt range ±60°, completeness 90.8%, resolution 0.71, independent reflections 342). Structure solution from the dataset Cr3_HH266, was possible despite the presence of additional reflections from the nolanite-type phase. Subsequent refinement of datasets Cr4_HH266 and Cr3_HH266 led to *R*(obs) values of 24.31% and 30.95%, respectively, taking only the kinematical approximation into account. Taking dynamical scattering into account led to improved *R*(obs) values of 14.52% and 11.72% (Table 3 ▸).

### 3.4. Powder X-ray diffraction

The synchrotron PXRD patterns show reflections of several not yet reported phases, which could be indexed. One of these phases is the novel nolanite-type phase, which could be detected in small quantities in the PXRD patterns of samples #HH228 and #HH266, shown in Figs. S2 and S3. In the PXRD pattern of #HH266, additional reflections consistent with the 6*R* polytype could be observed (Fig. S3). The unit-cell parameters were refined using a Le Bail fit (Table 4 ▸). The PXRD pattern of sample #HH670, which was synthesized at HP-HT conditions similar to #HH266, but from different starting materials, shows a large amount of the novel nolanite-type phase, together with a mixture of rutile-type SnO_2_ and GeO_2_, and spinel-type Ge_3_N_4_ (Fig. S4), which are well known phases. Therefore, a Rietveld refinement was possible for this sample. The resulting unit-cell parameters are included in Table 4 ▸.

### 3.5. Structure description of the nolanite-type phase

**Figure 6:**
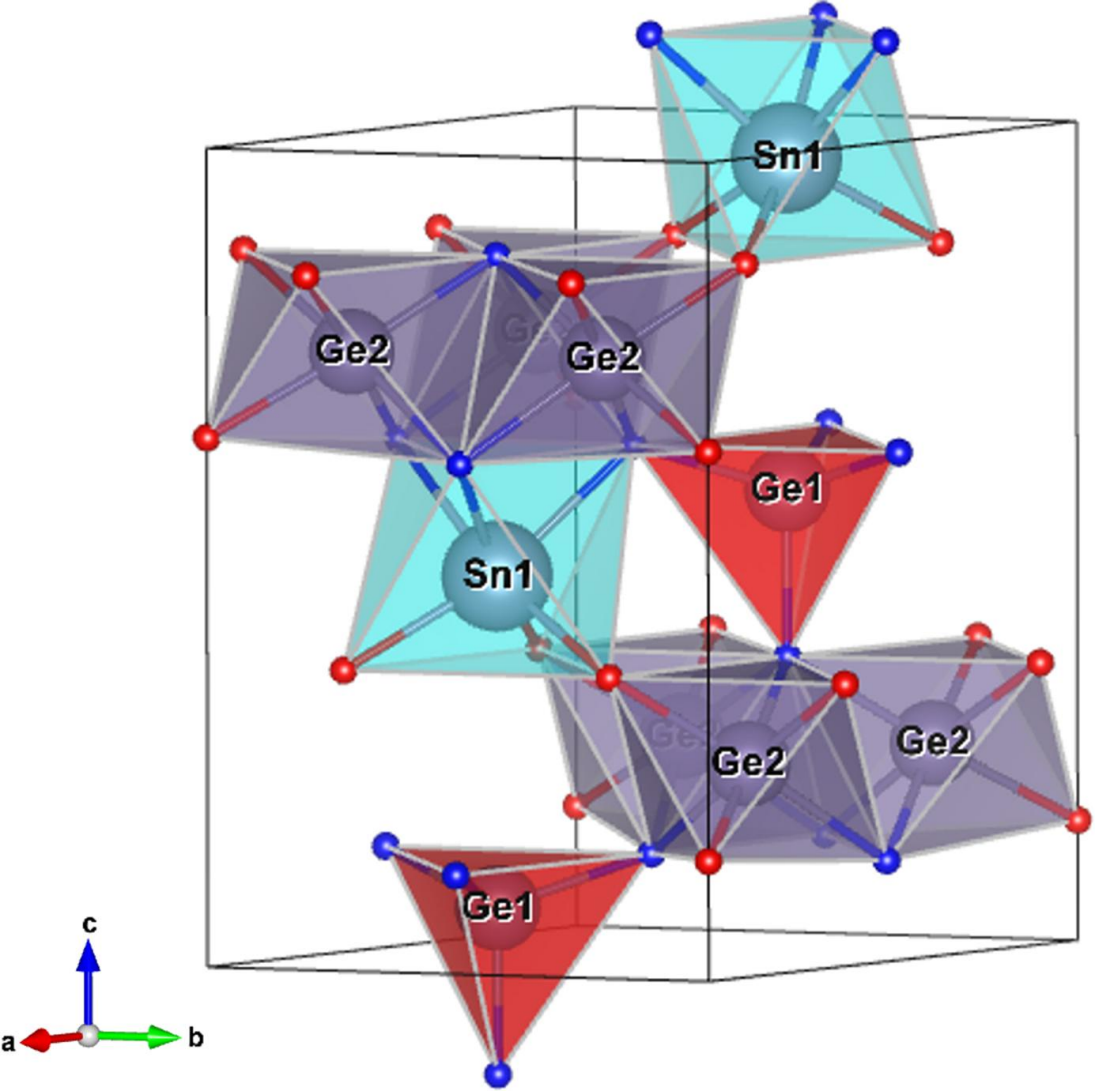
Crystal structure of nolanite-type SnGe_4_N_4_O_4_ showing the unit cell and the coordination polyhedra of the cations. O atoms are red, N atoms are blue. Picture generated with *Vesta* (Momma & Izumi, 2011 ▸).

The structure type found in Cr1_HH228 and Cr2_HH266 was attributed to the mineral nolanite (Hanson, 1958 ▸). In the crystal structure (Fig. 6 ▸), all atoms are located on mirror planes as indicated by the Wyckoff letters in Tables 1 ▸, 2 ▸ and 3 ▸. The anion sublattice is close-packed with an *ABAC* stacking sequence of the anions. Sn occupies an octahedral site, while Ge occupies both octahedral and tetrahedral sites. Interatomic distances for the nolanite-type phase can be found in supporting information. The cation-to-anion ratio is 5:8. Both germanium and tin have a valence of 4+, therefore charge neutrality can only be achieved by an N(3−):O(2−) = 1:1 ratio of the anions. As electron diffraction cannot distinguish between the light elements N and O in the presence of heavy metals, the N/O distribution on different sites has been adopted from the results of DFT calculations. The calculation of bond valence sums of the structure refinement further confirms the assignment of O and N (Table S16). All N atoms are fourfold coordinated and all O atoms are threefold coordinated. The N atoms are tetrahedrally coordinated by the cations, similar to the spinel structure, while the O atoms are coordinated in a trigonal pyramid.

**Figure 7:**
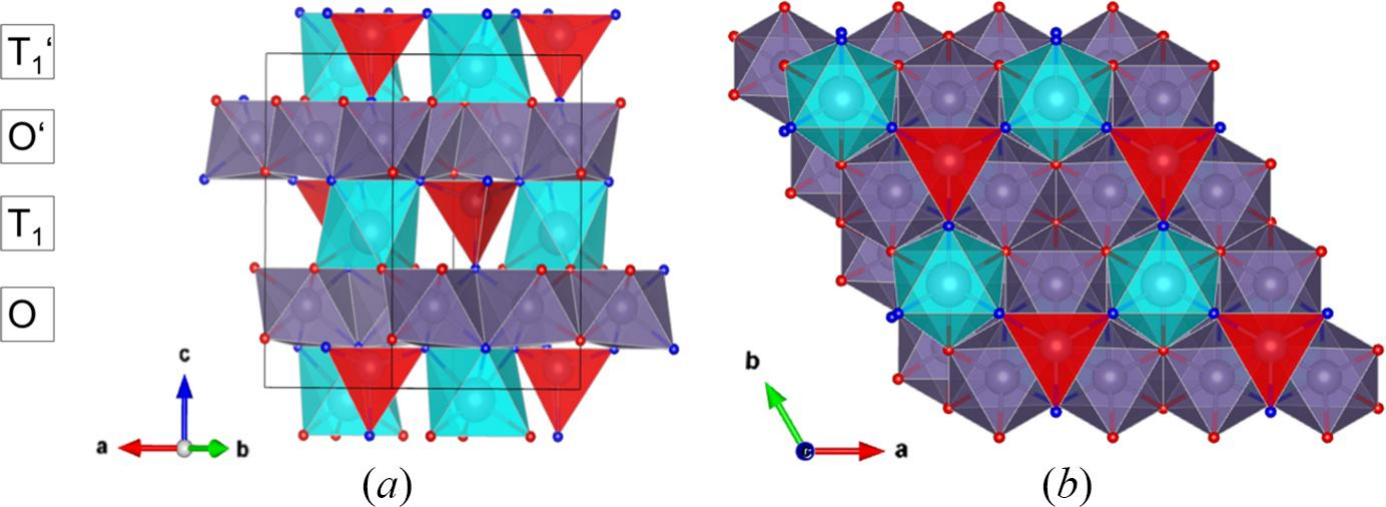
Structure of the nolanite-type phase viewed perpendicular to the *c*-axis (*a*) and along the *c*-axis (*b*). Ge1 tetrahedra are shown in red and Sn octahedra are shown in turquoise.

The structure is best described by considering layers perpendicular to the *c*-axis (Fig. 7 ▸). Two different types of layers can be distinguished. The first consists of octahedrally coordinated Ge on a 6*c* position and is called O-layer, according to the terminology of Grey & Gatehouse (1979 ▸). The second is a mixed T_1_-layer, containing tetrahedrally coordinated Ge next to octahedrally coordinated Sn on 2*b* positions. They are shown in Fig. 7 ▸(*a*) in an OT_1_O′T_1_′ sequence. O-layers are formed by edge-sharing GeN_3_O_3_ octahedra (Ge2 position), while the T_1_-layer is made up of corner-sharing GeN_4_ tetrahedra (Ge1 Position) and SnN_3_O_3_ octahedra, forming rings of alternating Sn- and Ge-centered polyhedra [Fig. 7 ▸(*b*)]. In addition, the SnN_3_O_3_ octahedra have edges in common with three GeN_3_O_3_ octahedra of an O-layer (via nitro­gen atoms) and share only corners with the GeN_3_O_3_ octahedra of the opposite O-layer (via oxygen atoms). The O′T_1_′-layers are related to the OT_1_-layers by a 180° rotation about the *c*-axis, provided by the 2_1_-screw axis (included in the 6_3_-screw axis). The relation of the pairs of layers can also be described by the *c*-glide plane.

#### 3.5.1. Structure description of the rhombohedral phase

The additional rhombohedral phase has the same chemical composition as the nolanite-type SnGe_4_N_4_O_4_. Oxygen and nitro­gen sites were assigned as given in the SnGe_4_N_4_O_4_ nolanite-type structure based on the same criteria, namely, fourfold-coordinated nitro­gen and threefold-coordinated oxygen. The coordination geometry stays identical. Interatomic distances can be found in supporting information. The structure consists of a close-packed anion lattice with cations in octahedral and tetrahedral sites, forming layers (O, T_1_) similar to the nolanite-type structure.

**Figure 8:**
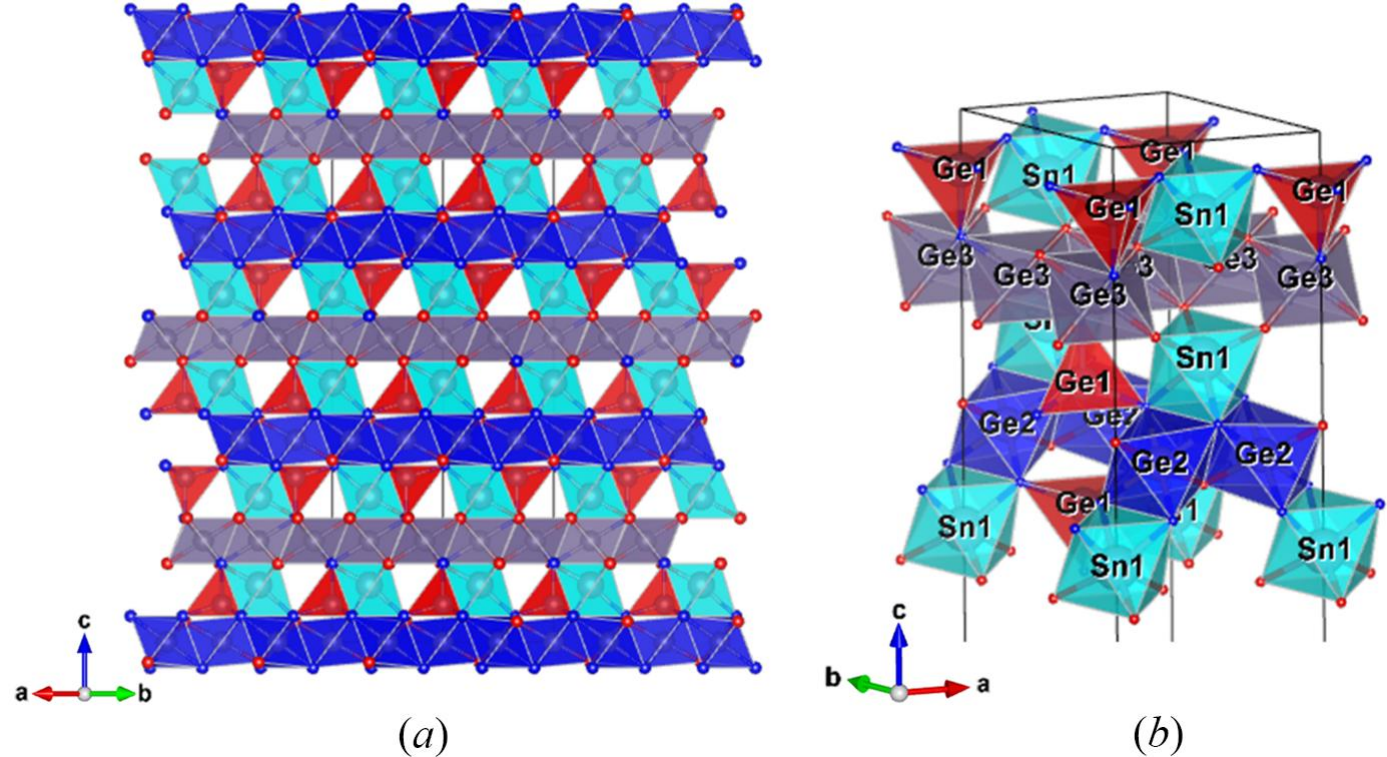
Structure of the rhombohedral phase, space group 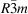 . Color code is the same as for the nolanite-type. Now there are two different non-equivalent O-layers formed by octahedra centered by Ge2 (shown in blue) and Ge3 (shown in gray). (*a*) Projection of the unit cell along [110], visualizing the polyhedron layers. (*b*) A part of the unit cell in perspective view, highlighting the connections between the different cation polyhedra in more detail.

T_1_-layers containing corner-sharing GeN_4_ tetrahedra and SnN_3_O_3_ octahedra, positioned on the threefold axis, form every second layer [Fig. 8 ▸(*a*)]. In between, there are two slightly different alternating octahedral (O) layers, formed by the symmetrically non-equivalent Ge2 and Ge3 atoms, which are both positioned on inversion centers (site symmetry 2/*m*). In every second T_1_-layer, the tetrahedra are oriented upside down along the *c*-axis [Figs. 8 ▸(*a*), 8 ▸(*b*)], described by the inversion center. Thus, the OT_1_O′T_1_′ block is slightly different from that of the nolanite type. Due to the difference of the two O-layers, the 6_3_-screw axis and the *c*-glide plane are lost. The two T_1_-layers instead are linked by the inversion center in the O-layers. This building block is tripled by the *R*-centering. According to the nomenclature for polytypes (Guinier *et al.*, 1984 ▸), the structure is a 6*R* polytype of the nolanite-type SnGe_4_N_4_O_4_.

**Figure 9:**
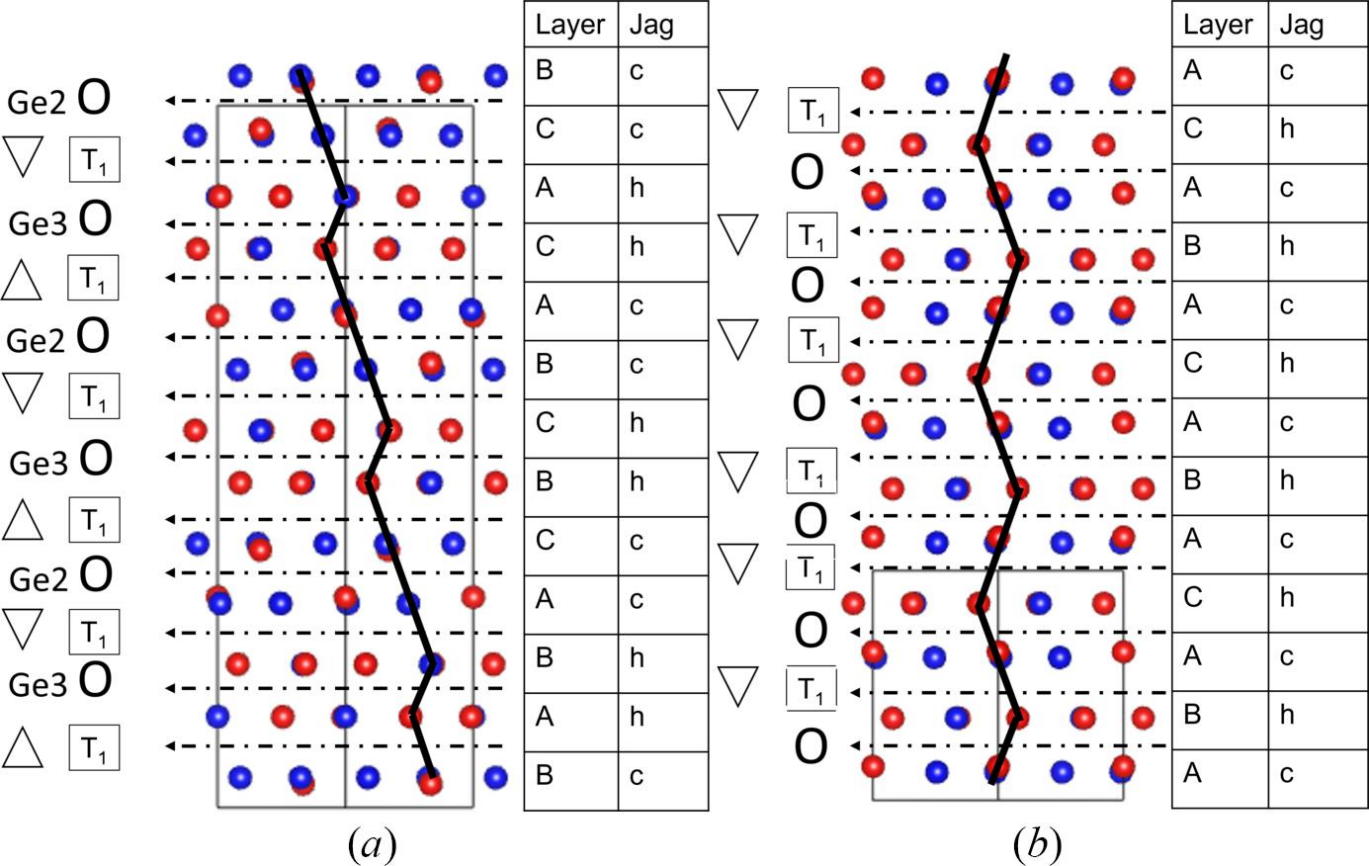
Comparison between the stacking of the anion layers of (*a*) the rhombohedral phase and (*b*) the basic nolanite type. The dotted arrows indicate the position of the cations. The O symbol denotes the presence of an O-layer and whether a Ge2 or a Ge3 atom is present. A triangle marks a T_1_-layer, with the direction of the tip of the triangle indicating the orientation of the GeN_4_ tetrahedron. Shown are the stacking vectors between the different layers and the resulting hexagonal (hcp) or cubic (ccp) close-packing sequences. In the legend (right-hand side), both the *ABC* and the Jagodzinski (1949 ▸) notation are given.

In the crystal structure of the 6*R* polytype the stacking sequence can be better understood by considering the stacking of the anion sublattice (Fig. 9 ▸). Both crystal structures show a mixture of cubic and hexagonal close packing. The nolanite structure has an *ABAC*-type stacking, which can be expressed as a c-h-c-h sequence using the notation by Jagodzinski (1949 ▸), where c denotes a layer with two different neighbors (cubic) and h one with two identical neighbors (hexagonal). For the 6*R* polytype, this sequence is 3 × (c-h-h-c). Each c-h-h-c sequence contains two T_1_-layers and one O-layer (Ge3), with an O-layer (Ge2) connecting the sequences (Fig. 8 ▸). A c-h-h-c sequence shifts by [2/3, 1/3, 1/3] from the one below, creating the rhombohedral centering.

### 3.6. Density functional theory: structure elucidation and site preference of anions and cations

Motivated by initial experimental data from 3D ED, nolanite-type structures of SnGe_4_N_4_O_4_ were explored, starting from data available for a magnesium molybdate [Inorganic Crystal Structure Database (ICSD) code: 248080; Abe *et al.* (2010 ▸)] with space group *P*6_3_ *mc* (space group No. 186, *Z* = 2). Simple valence rules suggest distributing O and N to three- and four-coordinated sites, respectively. The most straightforward pattern to distribute cations is to locate Sn in one of the twofold positions (Wyckoff 2*a*), resulting in either sixfold or fourfold coordination of tin. The structure with the lowest energy then comprises Sn with octahedral coordination, while Ge occupies the tetrahedral position and the remaining octahedral site (6*c*). To confirm the result, a series of calculations on alternative models was performed, with all of them leading to structures with higher energy. For example, switching O and N in Wyckoff positions 2*a* and 2*b* creates a model still in *P*6_3_ *mc* but with alternating layers of O and N. The energy difference to the lowest energy modification described above is about 1.5 eV per exchanged anion. The high value, which also reflects the costs of exchanging just a single O and N atom, indicates a low probability of finding significant O/N disorder among the anion sites. Similar energy differences are encountered in models with alternative distributions of cations. Placing Sn into the tetrahedral site, while keeping all germanium octahedrally coordinated, yields a model with a penalty of 1.5 eV per exchanged atom. Exchanging Sn and Ge among octahedral sites costs 1.0 eV per atom. Comparing the trends on Sn/Ge exchange supports the strong preference of Sn to occupy octahedral sites in the structure. The results provided feedback and input for further experimental refinement of the crystal structure.

### 3.7. Atomic resolution STEM

**Figure 10:**
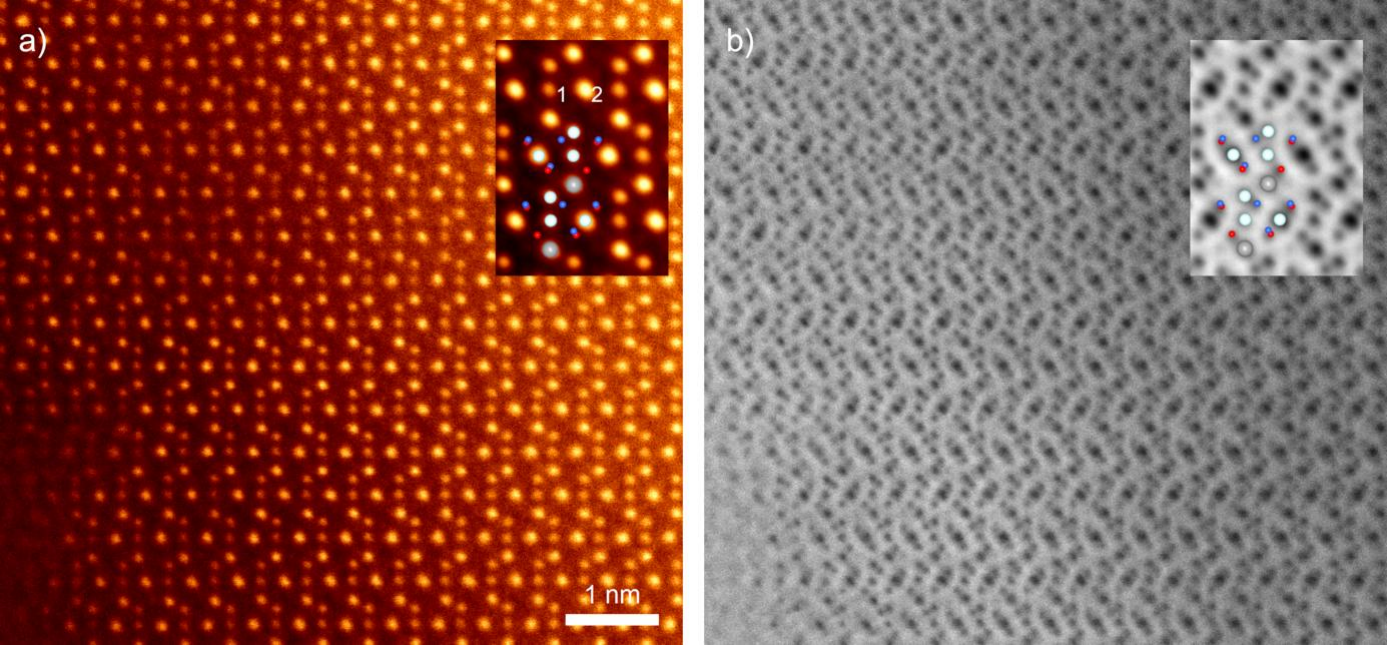
(*a*) ADF and (*b*) ABF atomic resolution STEM images of the nolanite-type phase along the [100] direction. The inset images are generated by unit-cell averaging, and the crystal structure model of Cr2_HH266 is overlaid. The metals are shown as larger balls (Sn gray, Ge light blue) and the anions as smaller balls (O red, N blue). The numbers in the left inset indicate a stacking of one or two germanium atoms per unit cell in the direction of observation.

As shown in Fig. 1 ▸(*b*), Ge-rich grains were found, which are expected to be SnGe_4_N_4_O_4_ with nolanite-type structure. Although the crystal structure was solved by ADT (see Section 3.3), the structure is complex. Therefore, it is essential to confirm the validity of the ADT refinement with a direct-space method, *i.e.* atomic resolution STEM imaging. Fig. 10 ▸(*a*) and Fig. 10 ▸(*b*) show the simultaneously acquired atomic resolution ADF and ABF STEM images, respectively, of the germanium-rich grain prepared by FIB milling. Owing to the *Z*-contrast nature (*Z* is the atomic number) in ADF STEM (Pennycook & Boatner, 1988 ▸), the cations of Ge and Sn are clearly visualized in Fig. 10 ▸(*a*). On the other hand, ABF STEM imaging has an excellent capability to visualize both heavy and light elements as dark dot contrast, including O and N (Findlay *et al.*, 2010 ▸). Combining ADF with ABF STEM imaging, it becomes possible to perform chemical-sensitive imaging of both light elements and heavy elements at an atomic scale. To enhance the signal-to-noise ratio, several tens of unit-cell images are averaged, and the images are given in the inset for the respective images (Ishikawa *et al.*, 2013 ▸). The bright atomic columns in Fig. 10 ▸(*a*) correspond to the Sn and Ge atomic columns. The brightness depends not only on the atomic number of the elements but also on the number of atoms in a column because the observation is in projection. For most of the positions, the stacking of atoms along the viewing direction is one atom per unit cell. At these positions, Sn appears brighter than Ge. Only in the octahedral layers, every second Ge atom is stacked with two atoms per unit cell, corresponding to the larger *Z*-contrast at that position [indicated by numbers 1 and 2 in the inset of Fig. 10 ▸(*a*)].

The structure model of Cr2_HH266 viewed along [100] is overlaid on the inset images, and all the anion and cation atomic columns are excellently matched with the ADT structure model. Therefore, it was directly confirmed that Sn is located not at the tetrahedral 2*b*, but at the octahedral 2*b* site.

## 4. Discussion

The novel phase is isostructural to the mineral nolanite reported by Hanson (1958 ▸) with the approximate composition Fe^2+^_2.5_V^3+^_1.5_V^4+^_6_O_16_. The octahedral positions in the O-layers are occupied exclusively by V^4+^, whereas the tetrahedra, as well as the octahedra of the T_1_-layers, are occupied by a mixture of Fe^2+^ and V^3+^, with an excess of the larger Fe ions in the octahedral position. Gatehouse *et al.* (1983 ▸) described a nolanite mineral with composition (VFeTiAl)_10_O_14_(OH)_2_. They found only V^3+^ and assigned the tetrahedral position to Fe alone. The remaining Fe excess, together with V, Ti and Al, was assumed to be distributed on the octahedral sites. They were unable to determine the distribution of these elements in the octahedral sites. In the nolanite-type mineral rinmanite (Zn, Mn, Mg)_2_(Sb)_2_(Fe,Mg)_6_O_14_(OH)_2_ (Holtstam *et al.*, 2001 ▸), the octahedra of the O-layers are shared by Mg^2+^ and Fe^3+^, whereas Sb^5+^ exclusively occupies the octahedra of the T_1_-layers (with Sb–O distances of 1.972 Å and 2.022 Å) and the tetrahedra contain a mixture of Zn^2+^ with a little Mn^2+^ and Mg^2+^ (cation–O distances of 1.958 Å and 1.986 Å). The corresponding cation–anion distances in nolanite-type SnGe_4_N_4_O_4_ exhibit a much larger difference between the octahedral and tetrahedral sites of the T_1_-layer. The average interatomic distances of 1.89 Å for the tetrahedrally coordinated Ge and 2.11 Å for the octahedral Sn further support the presence of Sn in the octahedral position.

Most of the synthetic nolanite-type compounds found in Pearson’s Crystal Data (Villars & Cenzual, 2024 ▸) are molybdates with composition *A*_2_Mo_3_O_8_, where tetravalent Mo occupies the octahedrally coordinated 6*c* position of the O-layers. The *A* cation occupies both positions in the T_1_-layer, where *A* may be a single element such as Mg, Mn, Fe, Co, Ni, Zn or Cd (McCarroll *et al.*, 1957 ▸), or a mixture of two elements as in (Sc,Zn)_2_Mo_3_O_8_ (Torardi & McCarley, 1985 ▸), (Li,Sc)_2_Mo_3_O_8_ (Kerner-Czeskleba & Tourne, 1976 ▸) and (InLi)_2_Mo_3_O_8_ (Kerner-Czeskleba & Tourne, 1976 ▸). In these compounds, the site preference in the T_1_-layer has not been investigated, or the close similarity of the corresponding ionic radii does not indicate a site preference. Rinmanite and nolanite are the only known isostructural compounds where the two positions in the T_1_-layer are occupied by different atoms.

Other examples, related to the nolanite-type structures, are the minerals from the nigerite group, which are polysomatic intergrowths of nolanite and spinel modules (Armbruster, 2002 ▸; Hejny & Armbruster, 2002 ▸; Grey & Gatehouse, 1979 ▸). In these minerals, Sn^4+^ occupies exclusively the octahedral positions of the T_1_-layer of the nolanite modules, showing the same preference as Sn^4+^ in the nolanite-type SnGe_4_N_4_O_4_. No nitro­gen-containing nolanite-type structures are reported in the Pearson database or the ICSD.

### 4.1. Description of the anion sublattice

To better understand both polytypes, considering the stacking of the anion sublattice can be helpful. The nolanite structure type and the 6*R* polytype differ in the stacking sequence of the anion layer. In both structures, the anion layers along **c** contain either three nitro­gen and one oxygen or three oxygen and one nitro­gen. In the nolanite type, a nitro­gen-rich layer is always followed by an oxygen-rich layer, while in the 6*R* polytype, two nitro­gen-rich layers are followed by two oxygen-rich layers (Fig. 9 ▸). This leads to the presence of two different O-layers, where one consists of GeN_4_O_2_ octahedra which are connected to the T_1_-layers via edge sharing and the other of GeN_2_O_4_ octahedra connected to the T_1_-layers via corner sharing. A nitro­gen-rich layer connects the O-layer and the octahedra of the T_1_-layer via edge sharing while an oxygen-rich layer does this via corner sharing.

This follows the observation from Grey & Gatehouse (1979 ▸) for the högbomite mineral series that for a c-c sequence (Jagodzinski notation) the O-layer is connected to the T_1_-layers via edge-sharing, while for an h-h sequence, it is via corner-sharing. In Fig. 9 ▸ it can be seen that for both the 6*R* polytype and the nolanite type, the oxygen-rich layers have the Jagodzinski symbol h (are hexagonal close-packed), while the nitro­gen-rich layers have the symbol c (are cubic close-packed).

One example is the mineral group högbomite built up from an anion sublattice with both hexagonal and cubic close packed stacking sequences, which is characterized by polysomatic intergrowths of modules of nolanite and spinel (Armbruster, 2002 ▸). The nolanite modules allow the incorporation of cations with different ionic radii and different valence into the structure. In the cubic close-packed modules of the 6*R* polytype, no additional tetrahedral gap is occupied and, therefore, no spinel-like T_2_-layer is present. Thus, the nominal ratio of tin to germanium remains the same (1:4) as in the nolanite-type structure. Accordingly, it seems that the appearance of the 6*R* polytype is due to the ordering of the anion layers and not due to the presence of additional cations. This is different compared to the högbomite minerals where the cubic close-packed and the hexagonal close-packed O-layers contain different cations. This shows that for oxynitride compounds based on close-packing, different stacking sequences of the anion layers are possible, allowing the formation of polytypes.

### 4.2. Comparison of results from different samples #HH228, #HH266 and #HH670

The two samples #HH228 (∼20 GPa, ∼1500°C, preparation under air) and #HH266 (∼16 GPa, ∼1200°C, preparation in a glovebox) have been synthesized at considerably different pressures and temperatures from an amorphous single-source precursor. This precursor consists of a homogeneous mixture of Sn, Ge, O and N at the atomic scale. The nolanite-type phase has been identified in the oxygen-exposed sample #HH228 and in the sample #HH266 which was protected from oxygen. From a qualitative comparison of the PXRD patterns of both samples it can be seen that #HH266 contains a higher amount of the nolanite-type phase (Figs. S2 and S3). The samples exhibit strong differences in the remaining phases present. α-PbO_2_-type SnO_2_ was not detected in #HH266, only in #HH228, most likely due to additional oxygen present in the sample. The investigation of the nolanite-type phase from both samples showed some small, but notable differences. The nolanite-type phase synthesized at higher pressure and temperature (#HH228) has a unit-cell volume of *V* = 281.6 Å^3^, compared to *V* = 276.5 Å^3^ for the nolanite-type phase from #HH266. The chemical composition derived from EDX is Sn: Ge = 1:4 in #HH228, which corresponds to the ideal stoichiometric composition SnGe_4_N_4_O_4_. For the crystals from #HH266, the EDX measurements on average show a smaller ratio of Sn:Ge = 0.75:4, which correlates with the smaller unit-cell volume. This can be explained by a partial replacement of Sn by Ge. An occupancy refinement of the cation positions of the nolanite structure from the dataset Cr2_HH266 confirmed this result with additional Ge replacing some Sn on the octahedral position leading to a ratio of Sn: Ge of around 0.8:4 (Table 2 ▸). Furthermore, in the PXRD of sample #HH266 the 6*R* polytype was detected (Fig. S3), which is not present in the PXRD of #HH228.

**Figure 11:**
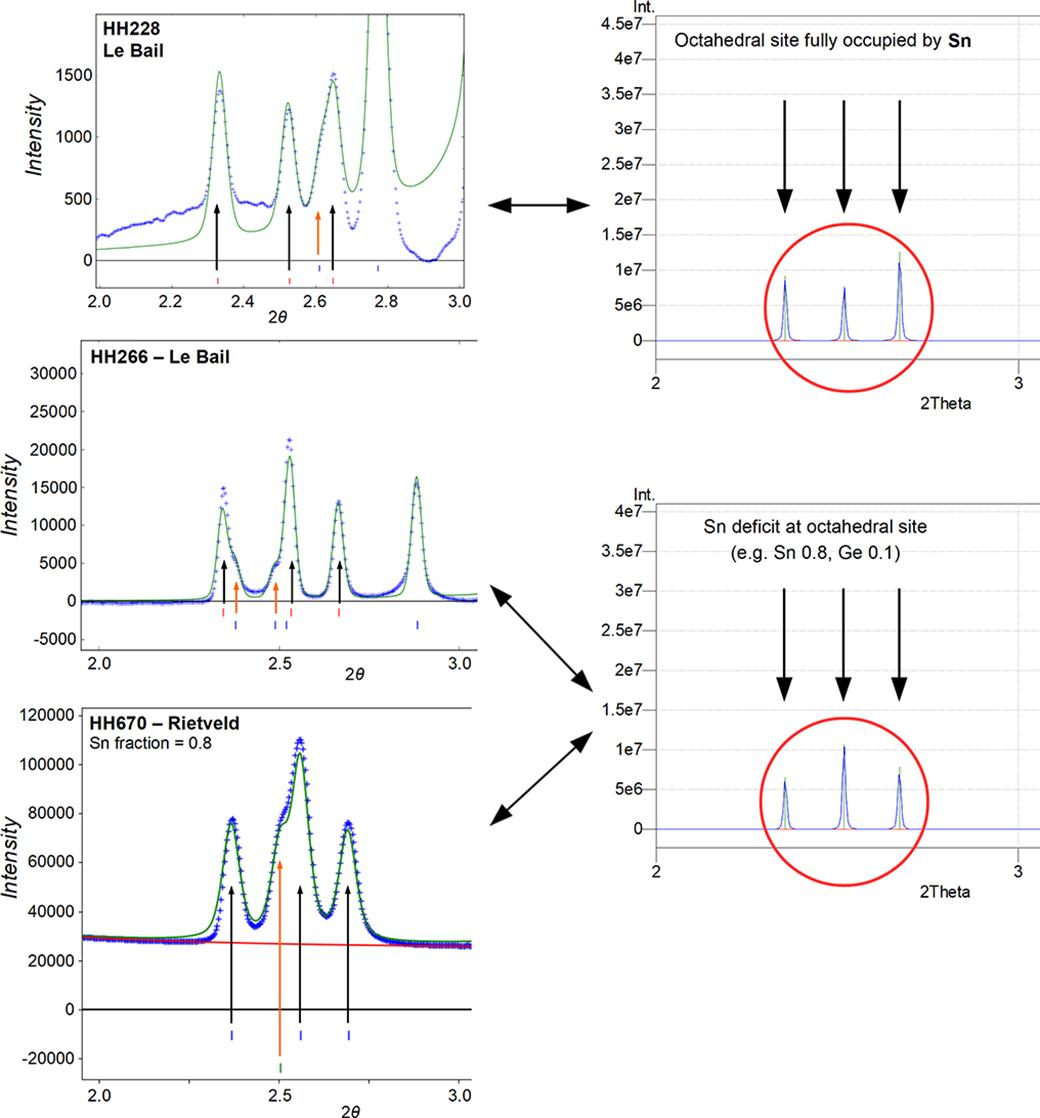
Comparison of the three low-angle reflections (marked by black arrows) of SnGe_4_N_4_O_4_ from different samples. These reflections are only marginally affected by overlap with other phases (indicated by orange arrows). Left-hand column: synchrotron powder XRD patterns (λ = 0.207 Å) as measured. Right-hand column: powder patterns simulated from the structure model (atomic positions from DFT results). Only for sample #HH228 the relative intensity of these three reflections corresponds to the simulation which assumes the octahedral site is fully occupied by Sn. For the other samples, #HH266 and #HH670, the relative intensity qualitatively corresponds to a simulation with a certain Sn deficit. This supports the idea that the lower unit-cell volume of crystals from #HH266 and #HH670, as compared to #HH228, is caused by a slightly lower Sn content.

To verify the results from samples #HH228 and #HH266 another synthesis was made, using a different precursor material. Sample #HH670 was prepared from a mechanical mixture of crystalline phases, namely, rutile-type SnO_2_ and sinoite-type Ge_2_N_2_O matching the chemical composition of SnGeN_4_O_4_. For comparison, the sample was synthesized at the same P/T conditions as #HH266. The resulting different phase composition is attributed to the different starting materials. The crystalline precursor powder is a less homogenous starting material in comparison to that of the polymeric single source precursor derived material. Therefore, while #HH670 contains a large amount of the novel nolanite-type phase, it contains significant amounts of unreacted rutile-type SnO_2_ and left-over Ge in the form of rutile-type GeO_2_ and spinel-type Ge_3_N_4_. The unit-cell volume *V* = 269.4 Å^3^ of the nolanite-type phase in sample #HH670 is even smaller in comparison to the other samples, suggesting an even larger Sn deficit. This was confirmed by the Rietveld refinement of sample #HH670, which resulted in a Sn occupancy of about 0.8, corresponding to a Sn:Ge ratio of 0.8:4. A partial replacement of Sn by Ge could not be quantified from the PXRD data. Qualitatively, the varying Sn occupancy in the different samples is clearly visible in the PXRD patterns by the variation of the relative intensity of some low-angle reflections, as compared to simulated powder patterns (Fig. 11 ▸). The fact that the new phase has been synthesized using two different routes suggests that the nolanite-type phase is indeed thermodynamically stable, at least under high-pressure conditions.

## 5. Conclusion

In this study a new nolanite-type mixed Sn Ge oxynitride was synthesized under high-pressure high-temperature conditions. The crystal structure was solved via ADT, a 3D ED method and characterized with a combination of PXRD, EDX, 3D ED, DFT calculation and atomic resolution STEM. This phase is the first nolanite-type oxynitride, as well as the first mixed Sn Ge oxynitride phase reported in the literature. The crystal structure is made up of a close-packed anion lattice with Ge being present in both tetrahedral and octahedral coordination and Sn in octahedral coordination. DFT calculations suggested that the O and N atoms are not randomly distributed on the anion positions, but instead occupy distinct positions. Using a variety of different methods, it could be established that the Sn occupies the octahedral position in the T_1_-layer, while the Ge occurs in both tetrahedral and octahedral coordination. This is similar to Ge_3_N_4_, which under high-pressure conditions forms the spinel structure where the Ge shows both tetrahedral and octahedral coordination (Leinenweber *et al.*, 1999 ▸). The octahedral coordination of Sn is in agreement with high-pressure studies of SnO_2_ (Haines & Léger, 1997 ▸; Ono *et al.*, 2005 ▸).

Differences in the unit-cell parameters from PXRD between the different samples and the different results from EDX measurements suggest that up to 20% of the Sn can be replaced by germanium.

In addition to the nolanite-type structure, in sample #HH266 a 6*R* polytype of the nolanite-type phase with a more complex stacking order could be identified and its crystal structure was solved and refined.

In conclusion, it is possible to produce mixed SnGe oxynitride compounds *via* high-pressure high-temperature synthesis. This opens pathways for new compounds with interesting properties and might shed further light on the behavior of Sn and Ge compounds under high-pressure conditions.

## 6. Related literature

The following references, not cited in the main body of the paper, have been cited in the supporting information: Brese & O’Keeffe (1991 ▸); Brown (2020 ▸); Gagné & Hawthorne (2015 ▸).

## Supplementary Material

Crystal structure: contains datablock(s) global, Cr1_HH228, Cr2_HH266, Cr3_HH266, Cr4_HH266. DOI: 10.1107/S2052520624002683/je5053sup1.cif

Structure factors: contains datablock(s) Cr1_HH228. DOI: 10.1107/S2052520624002683/je5053Cr1_HH228sup2.hkl

Structure factors: contains datablock(s) Cr2_HH266. DOI: 10.1107/S2052520624002683/je5053Cr2_HH266sup3.hkl

Structure factors: contains datablock(s) Cr3_HH266. DOI: 10.1107/S2052520624002683/je5053Cr3_HH266sup4.hkl

Structure factors: contains datablock(s) Cr4_HH266. DOI: 10.1107/S2052520624002683/je5053Cr4_HH266sup5.hkl

Tables S1-S18 and Figs. S1-S7. DOI: 10.1107/S2052520624002683/je5053sup6.pdf

CCDC references: 2335472, 2335473, 2335474, 2335475

## Figures and Tables

**Table 1:** Atomic coordinates, displacement parameters and symmetry information of the nolanite-type SnGe_4_N_4_O_4_ structure (space group *P*6_3_ *mc*) resulting from the dynamical refinement of ADT data of crystal Cr1_HH228

	*x*	*y*	*z*	*U*_equiv_	Wyckoff letter	Site symmetry
Sn1	0.333333	0.666667	0.9666 (3)	0.0070 (4)	2*b*	3*m*.
Ge1	0.333333	0.666667	0.5613 (3)	0.0052 (6)	2*b*	3*m*.
Ge2	−0.16655 (18)	0.16655 (18)	0.7463 (3)	0.0052 (4)	6*c*	.*m*.
N1	0.333333	0.666667	0.3579 (6)	0.007 (2)	2*b*	3*m*.
N2	−0.4900 (5)	0.4900 (5)	0.6219 (5)	0.0053 (15)	6*c*	.*m*.
O1	0	0	0.6465 (7)	0.0030 (17)	2*a*	3*m*.
O2	0.1569 (6)	−0.1569 (6)	0.8573 (4)	0.0105 (18)	6*c*	.*m*.

**Table 2:** Atomic coordinates, displacement parameters and symmetry information of the nolanite-type SnGe_4_N_4_O_4_ structure (space group *P*6_3_ *mc*) resulting from the dynamical refinement of ADT data of crystal Cr2_HH266

	*x*	*y*	*z*	*U*_iso_	Occupancy	Wyckoff letter	Site symmetry
Sn1	0.333333	0.666667	0.9648 (6)	0.0032 (6)	0.84 (3)	2*b*	3*m*.
Ge(Sn1)	0.333333	0.666667	0.9648 (6)	0.0032 (6)	0.16 (3)	2*b*	3*m*.
Ge1	0.333333	0.666667	0.5626 (8)	0.0080 (8)	1	2*b*	3*m*.
Ge2	−0.16716 (17)	0.16716 (17)	0.7464 (6)	0.0032 (4)	1	6*c*	.*m*.
N1	0.333333	0.666667	0.3558 (17)	0.006 (3)	1	2*b*	3*m*.
N2	−0.4899 (5)	0.4899 (5)	0.6231 (11)	0.0038 (11)	1	6*c*	.*m*.
O1	0	0	0.6486 (15)	0.0013 (18)	1	2*a*	3*m*.
O2	0.1541 (6)	−0.1541 (6)	0.8585 (11)	0.0105 (14)	1	6*c*	.*m*.

**Table 3:** Atomic positions, displacement parameters, and symmetry information of the 6*R* polytype (space group 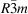 ) resulting from the dynamical refinement of ADT data of crystal Cr4_HH266

	*x*	*y*	*z*	*U*_iso_	Wyckoff letter	Site symmetry
Sn1	0	0	0.42713 (7)	0.0053 (6)	6*c*	3*m*
Ge1	0	0	0.06208 (11)	0.0081 (8)	6*c*	3*m*
Ge2	0.5	0	0	0.0080 (12)	9*e*	.2/*m*
Ge3	0.5	0	0.5	0.0065 (6)	9*d*	.2/*m*
O1	0	0	0.7003 (3)	0.0022 (18)	6*c*	3*m*
N1	0	0	0.1290 (3)	−0.0035 (17)	6*c*	3*m*
N2	0.9834 (13)	0.4917 (7)	0.7084 (2)	0.0055 (12)	18*h*	.*m*
O2	0.8447 (8)	0.1553 (8)	0.7962 (2)	0.0188 (16)	18*h*	.*m*

**Table 4:** Unit-cell parameters of nolanite-type SnGe_4_N_4_O_4_ for different samples and the 6*R* polytype, derived from the synchrotron PXRD measurements, and from DFT calculations of the nolanite type

	*a* (Å)	*c* (Å)	*V* (Å^3^)
#HH228	5.876 (3)	9.418 (5)	281.6 (2)
#HH266	5.839 (1)	9.365 (2)	276.55 (7)
#HH670	5.791 (1)	9.276 (2)	269.38 (7)
6*R* polytype (#HH266)	5.846 (1)	28.230 (5)	835.5 (2)
DFT	5.818	9.427	276.4
