# Supplementary material for: Crystal structures of two new high-pressure oxynitrides with composition SnGe4N4O4, from single-crystal electron diffraction
Source: Acta Crystallogr B Struct Sci Cryst Eng Mater. 2024 May 8;80(Pt 3):182–92. doi: 10.1107/S2052520624002683 (PMC11157342; doi:10.1107/S2052520624002683)
Supplement: Supplementary file 6 [file b-80-00182-sup6.pdf]

Acta Cryst B

STRUCTURAL SCIENCE
CRYSTAL ENGINEERING
MATERIALS

Volume 80 (2024)

Supporting information for article:

Crystal structures of two new high-pressure oxynitrides with composition SnGe4N4O4, from single-crystal electron diffraction

Philipp Gollé-Leidreiter, Shrikant Bhat, Leonore Wiehl, Qingbo Wen, Peter Kroll, Ryo Ishikawa, Martin Etter, Robert Farla, Yuichi Ikuhara, Ralf Riedel and Ute Kolb

S1. Synthesis

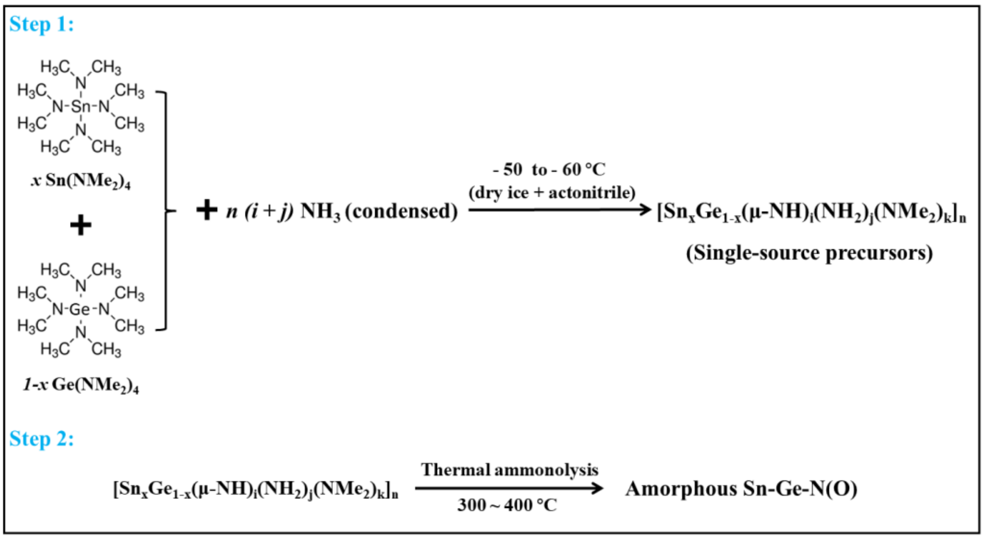

Figure S1 Details of the synthesis of the amorphous Sn-Ge-N(O) for the high-pressure high-temperature synthesis

S2. PXRD

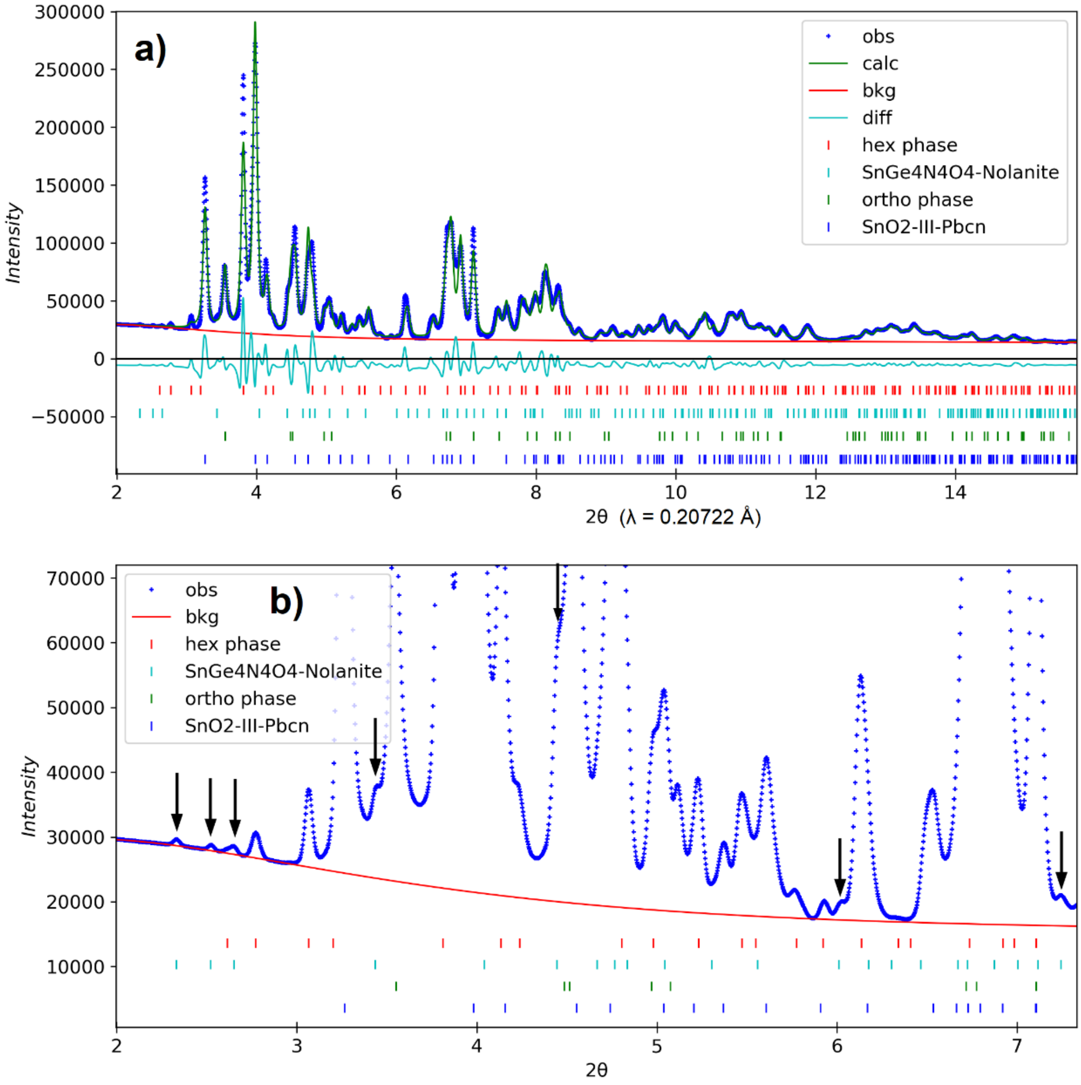

Figure S2 (a) Powder XRD pattern of sample **#HH228**, measured with synchrotron radiation (λ = 0.20722 Å), showing the Le Bail fit of a mixture of four different, partially known phases. Only a small fraction of the novel nolanite-type $SnGe_4N_4O_4$ ($P6_3mc$) is contained in this sample and the tiny reflections are scarcely visible in the full pattern. (b) Magnified section of the XRD pattern, highlighting $SnGe_4N_4O_4$ reflections, which show no overlap with other phases (black arrows). These reflections have been used to determine the lattice parameters of this phase, namely a = 5.876(3) Å and c = 9.418(5) Å.

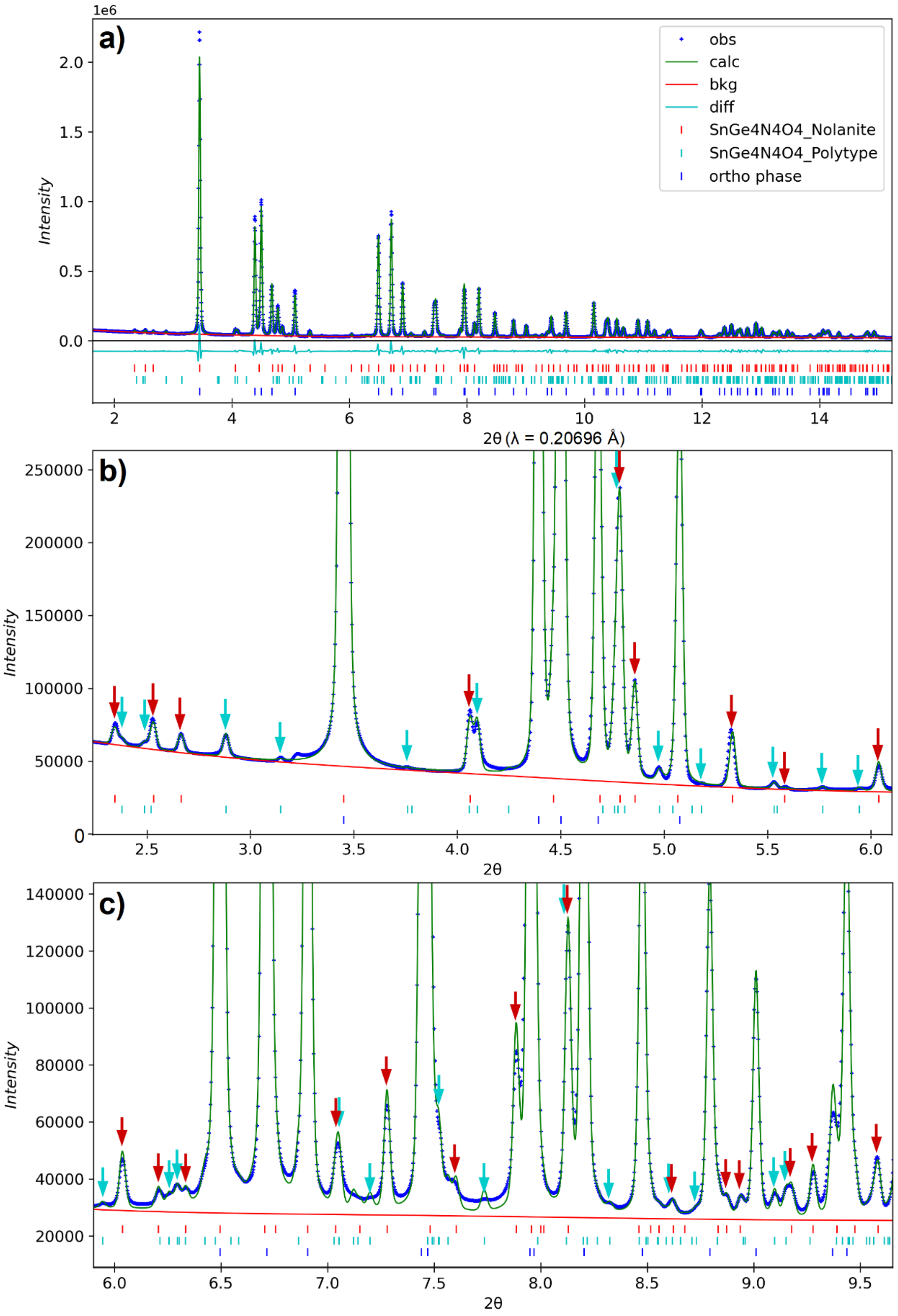

Figure S3 Synchrotron powder XRD pattern of sample **#HH266** with Le Bail fit (wR = 5.7 %) of three phases, namely nolanite-type $SnGe_4N_4O_4$ ($P6_3mc$), the 6R polytype of $SnGe_4N_4O_4$ ($R\overline{3}m$) and an orthorhombic phase with still unknown structure. (a) Full pattern, dominated by the orthorhombic

phase. (b, c) Magnified sections of the pattern in the angle range up to 2θ = 6° and from 6° to 9.5°, respectively. The arrows indicate reflections of the nolanite-type phase (red) and the polytype (cyan), which show no overlap with the ortho phase. Only such reflections have been used to determine the lattice parameters of the two phases, namely *a* = 5.839(1) Å, *c* = 9.365(2) Å for the nolanite type phase, and *a* = 5.846(1) Å, *c* = 28.230(5) Å for the polytype.

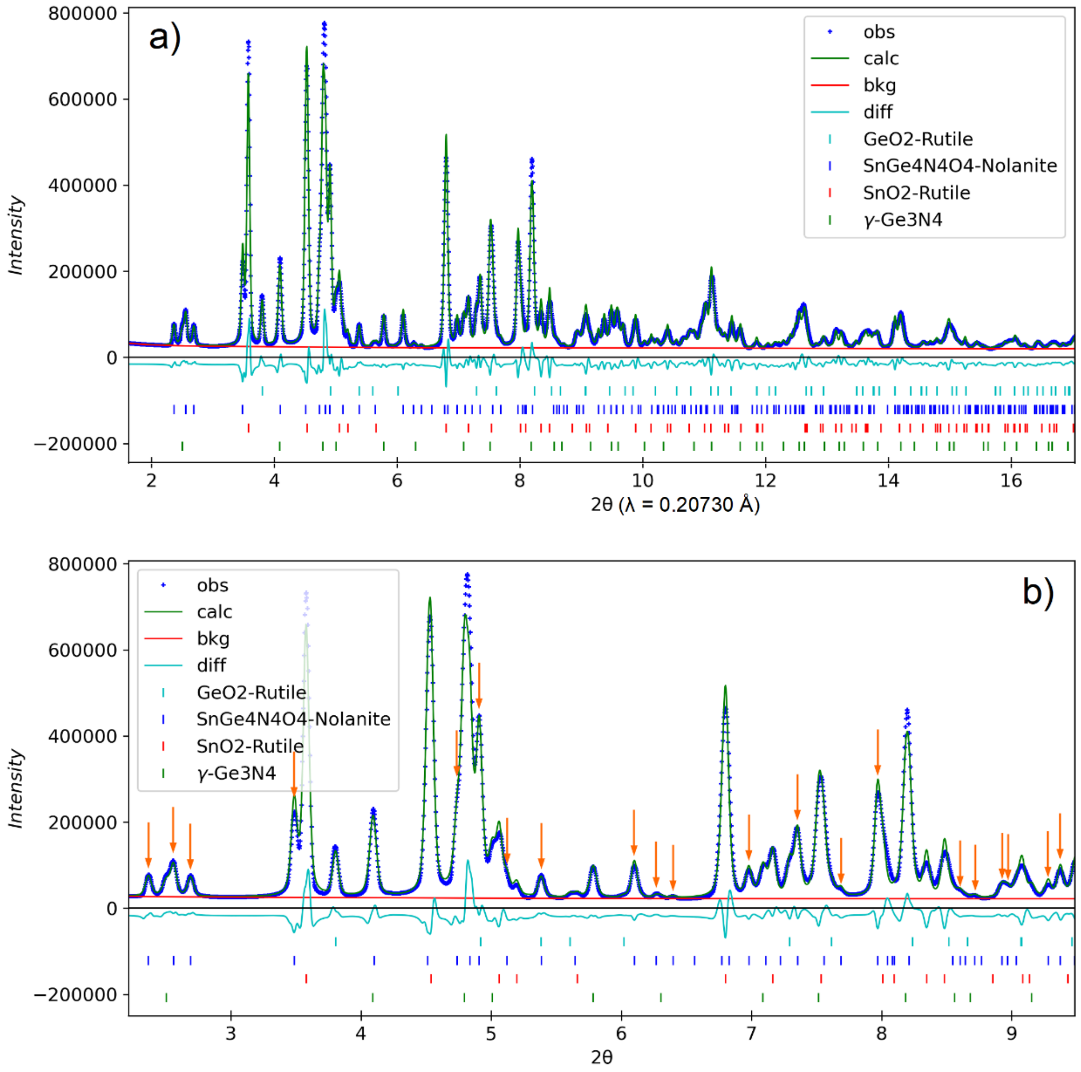

Figure S4 (a) Synchrotron powder XRD pattern of sample **#HH670** with Rietveld refinement (wR = 11.0 %) of nolanite-type $SnGe_4N_4O_4$ ($P6_3mc$, 41.0 wt%) and three other known phases, namely spinel-type γ-Ge_3N_4 (*Fd*-3*m*, 28.9 wt%) and rutile-type ($P4_2/mnm$) SnO_2 (24.1 wt%) and GeO_2 (6.0 wt%). (b) Magnified section of the pattern in the angle range, up to 2θ = 9.5°. The orange arrows indicate reflections of the nolanite-type phase, which show no overlap with other phases. The refined lattice parameters of nolanite-type $SnGe_4N_4O_4$ are *a* = 5.791(1) Å, *c* = 9.276(2) Å.

Table S1 Overview of the samples investigated in this work. SnO_2-III denotes the PbO_2-type polymorph, while SnO_2-I/GeO_2-I denotes the rutile-type polymorph.

Sample	Pressure (GPa)	Temperature (°C)	Precursor	Preparation of the capsule	Quantity of nolanite-type phase	Other phases present
#HH228	20	1500	Polymer derived	Under air	Minor	SnO_2-III, other novel phases (analysis in progress)
#HH266	16	1200	Polymer derived	Glovebox	Small	Other novel phases (analysis in progress)
#HH670	15.6	1200	Crystalline powder of 67 mol% Ge_2N_2O and 33 mol% SnO_2-I	Glovebox	41wt%	Spinel type Ge_3N_4 (28.9 wt%), SnO_2-I (24.1 wt%) GeO_2-I (6wt%)

S3 EDX

S3.1 TEM-EDX

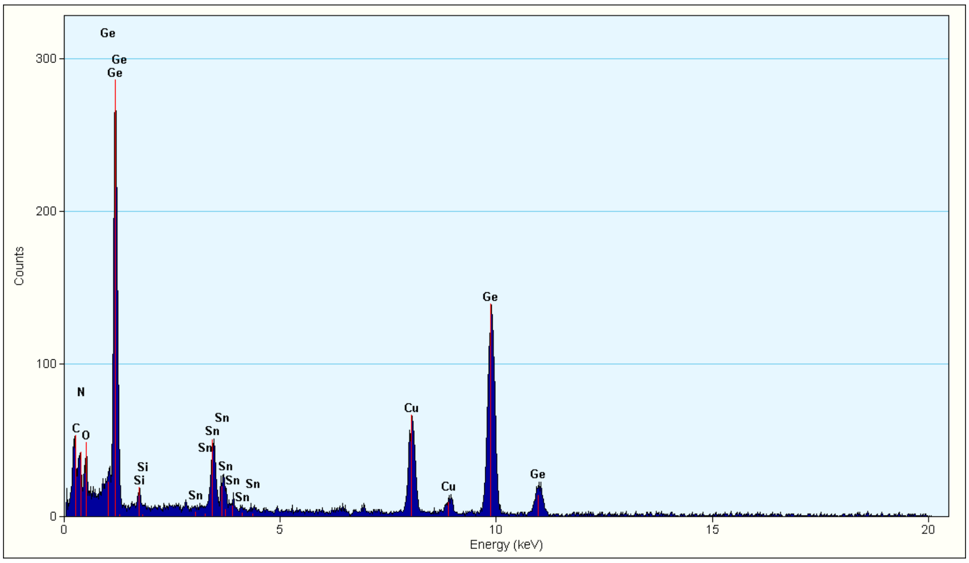

Figure S5 EDX spectrum of Cr1_HH228. C and Cu are contributions from the TEM grid.

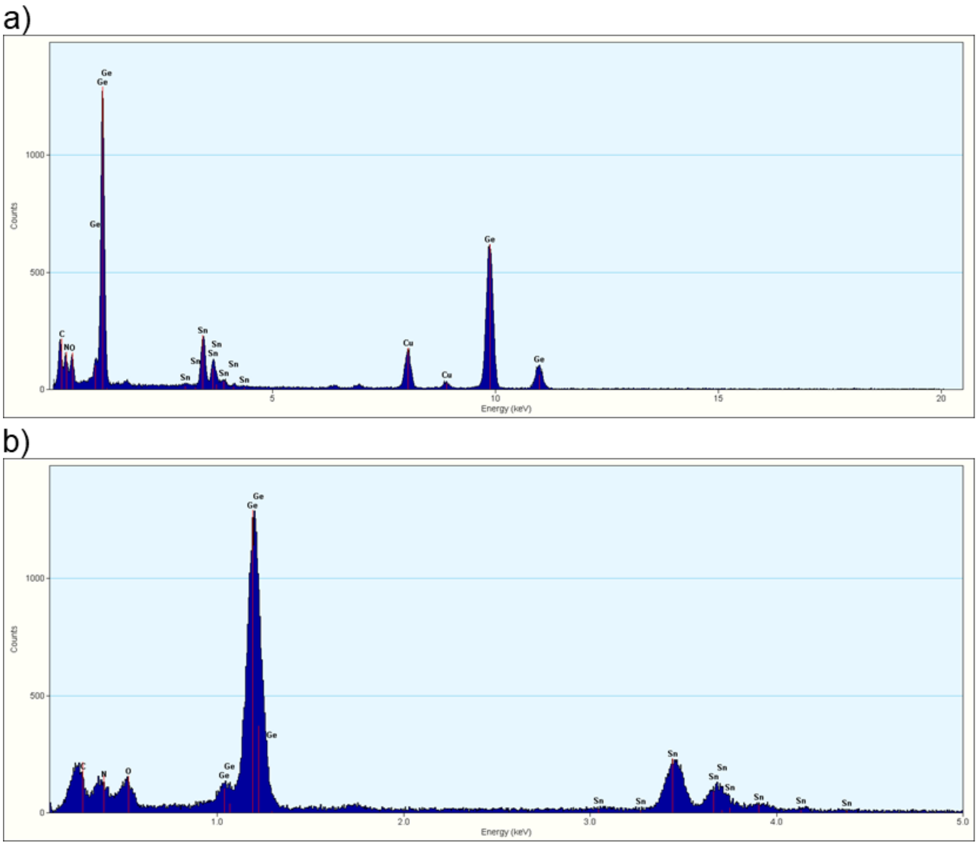

Figure S6 EDX Spectrum of the nolanite phase from HH266, a) full spectrum, b) magnified spectrum up to 6 keV. C and Cu peaks are from the TEM grid.

S4 ADT

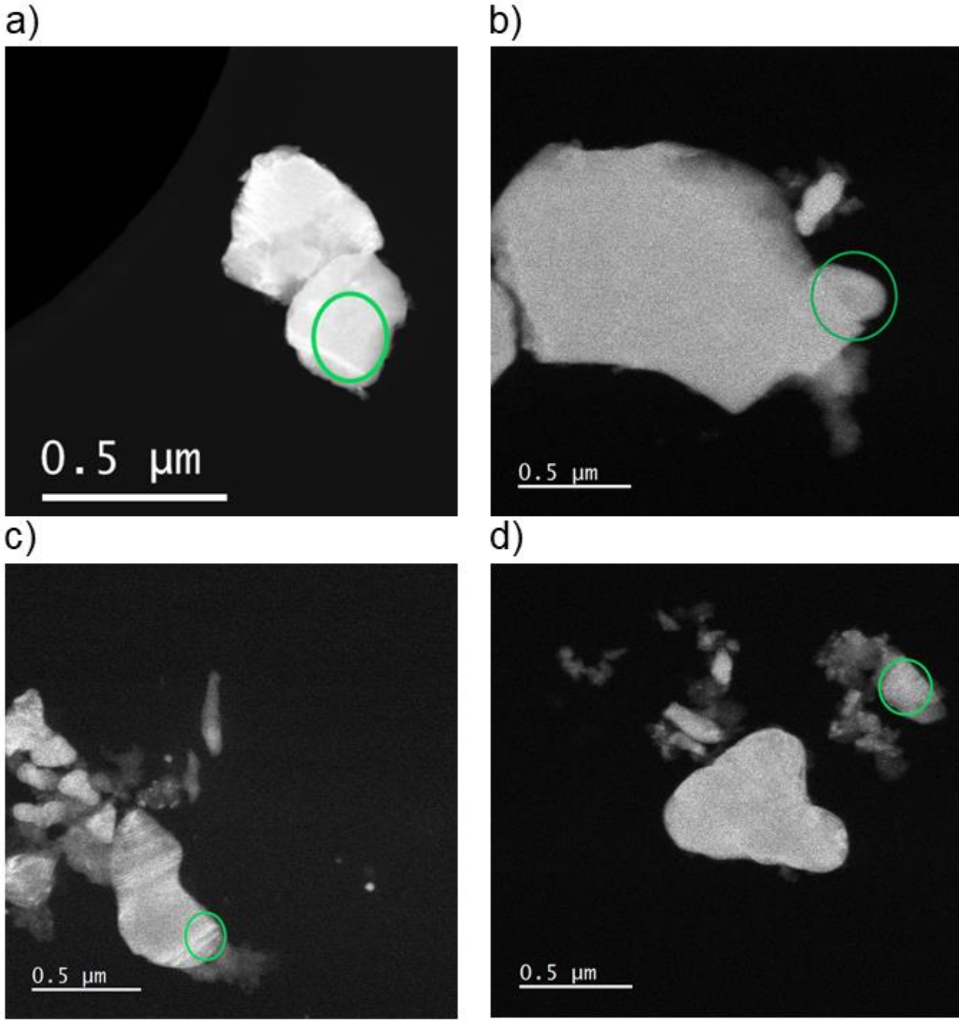

Figure S7 HAADF-STEM Pictures of the spots where ADT data was acquired. The green ring marks the position of the beam a) Cr1_HH228 b) Cr2_HH266 c) Cr3_HH266 d) Cr4_HH266.

Table S2 Atomic positions, displacement parameters, and symmetry information of the 6*R* poly-type, space group $R\overline{3}m$, resulting from the dynamical refinement of ADT data of crystal Cr3_HH266.

	x	*y*	*z*	U_{iso}	Wyckoff letter	Site symmetry
Sn1	0	0	0.42707(9)	-0.0006(7)	6*c*	3*m*
Ge1	0	0	0.06196(13)	0.0034(9)	6*c*	3*m*
Ge2	0.5	0	0	-0.0013(7)	9*e*	.2/*m*
Ge3	0.5	0	0.5	-0.0036(7)	9*d*	.2/*m*
O1	0	0	0.7003(3)	-0.002(2)	6*c*	3*m*

N1	0	0	0.1297(3)	-0.0189(16)	6c	3*m*
N2	0.9793(14)	0.4897(7)	0.7092(2)	-0.0002(15)	18h	.*m*
O2	0.8427(7)	0.1573(7)	0.7956(2)	0.0059(15)	18h	.*m*

Table S3 Interatomic anion-cation distances of Cr1_HH228, $P6_3mc$ after dynamical refinement.

Atom 1	Atom 2	Count	d 1,2 (Å)
Sn1	O2	3x	2.070(4)
	N2	3x	2.164(4)
Ge1	N2	3x	1.886(4)
	N1	1x	1.915(7)
Ge2	O1	1x	1.938(3)
	O2	2x	1.952(4)
	N1	1x	1.997(4)
	N2	2x	2.023(4)

Table S4 Interatomic cation-cation and anion-anion distances of Cr1_HH228, $P6_3mc$ after dynamical refinement.

Atom 1	Atom 2	Count	d 1,2 (Å)	Atom 1	Atom 2	Count	d 1,2 (Å)
Sn1	Ge2	3x	3.133(3)	N1	N2	3x	2.736(7)
	Ge1	3x	3.5076(10)		O2	6x	2.940(4)
	Ge2	6x	3.597(3)		N2	3x	3.068(7)
Ge1	Ge2	9x	3.416(2)	N2	N1	1x	2.736(7)
	Sn1	3x	3.5077(9)		N2	2x	2.762(6)
Ge2	Ge2	4x	2.938(2)		O2	2x	2.854(5)
	Sn1	1x	3.133(3)		O1	2x	2.949(2)
	Ge1	3x	3.416(2)		O2	2x	3.018(5)
	Sn1	2x	3.597(2)		N1	1x	3.068(7)
					N2	2x	3.114(6)

	O1	O2	3x	2.548(6)
		N2	6x	2.949(4)
		O2	3x	3.157(7)
	O2	O1	1x	2.548(6)
		O2	2x	2.765(6)
		N2	2x	2.854(5)
		N1	2x	2.940(4)
		N2	2x	3.018(5)
		O2	2x	3.111(6)
		O1	1x	3.157(7)

Table S5 Interatomic anion-cation distances of Cr2_HH266, $P6_3mc$ after dynamical refinement.

Atom 1	Atom 2	Count	d 1,2 (Å)
Sn1|Gesn1	O2	3x	2.069(7)
	N2	3x	2.169(8)
Ge1	N2	3x	1.875(5)
	N1	1x	1.936(2)
Ge2	O1	1x	1.923(7)
	O2	2x	1.937(7)
	N1	1x	1.969(9)
	N2	2x	2.001(7)

Table S6 Interatomic cation-cation and anion-anion distances of Cr2_HH266, $P6_3mc$.

Atom 1	Atom 2	Count	d 1,2 (Å)	Atom 1	Atom 2	Count	d 1,2 (Å)
Sn1|Gesn1	Ge2	3x	3.127(6)	N1	N2	3x	2.694(16)
	Ge1	3x	3.493(2)		O2	6x	2.922(4)
	Ge2	6x	3.565(5)		N2	3x	3.075(16)
Ge1	Ge2	9x	3.389(5)	N2	N1	1x	2.694(16)

	Sn1|Gesn1	3x	3.493(2)		N2	2x	2.742(6)
Ge2	Ge2	4x	2.928(2)		O2	2x	2.85(1)
	Sn1|Gesn1	1x	3.127(6)		O1	2x	2.931(3)
	Ge1	3x	3.389(4)		O2	2x	2.937(9)
	Sn1|Gesn1	2x	3.565(4)		N1	1x	3.075(2)
					N2	2x	3.097(6)
				O1	O2	3x	2.51(1)
					N2	6x	2.931(3)
					O2	3x	3.132(3)
				O2	O1	1x	2.51(1)
					O2	2x	2.699(7)
					N2	2x	2.85(1)
					N1	2x	2.922(4)
					N2	2x	2.937(9)
					O1	1x	3.132(3)
					O2	2x	3.140(7)

Table S7 Interatomic anion-cation distances of Cr3_HH266, $R\bar{3}m$ after dynamical refinement.

Atom 1	Atom 2	Count	d 1,2 (Å)
Sn1	O2	3x	2.042(6)
	N2	3x	2.144(7)
Ge1	N2	3x	1.874(7)
	N1	1x	1.911(9)
Ge2	O1	2x	1.936(5)
	N2	4x	2.031(7)
Ge3	O2	4x	1.957(5)
	N1	2x	1.985(4)

Table S8 Interatomic cation-cation and anion-anion distances of Cr3_HH266, $R\overline{3}m$.

Atom 1	Atom 2	Count	d 1,2 (Å)	Atom 1	Atom 2	Count	d 1,2 (Å)
Sn1	Ge2	3x	3.139(2)	N2	O1	1x	2.67(1)
	Ge1	3x	3.493(1)		N2	2x	2.74(1)
	Ge3	6x	3.575(1)		O1	2x	2.936(6)
Ge1	Ge3	3x	3.404(3)		O2	2x	2.969(8)
	Ge2	6x	3.406(2)		N2	2x	3.00(1)
	Sn1	3x	3.493(1)		N1	1x	3.043(9)
	Ge1	1x	3.498(5)		N2	2x	3.104(7)
Ge2	Ge2	4x	2.923	O2	N1	1x	2.643(9)
	Sn1	2x	3.139(2)		O2	2x	2.758(8)
	Ge1	4x	3.406(2)		O2	2x	2.776(8)
Ge3	Ge3	4x	2.923		N1	2x	2.925(3)
	Ge1	2x	3.404(3)		N2	2x	2.969(8)
	Sn1	4x	3.575(1)		O2	2x	3.088(8)
					O1	1x	3.13(1)
				O1	N2	3x	2.67(1)
					N2	6x	2.936(6)
					O2	3x	3.13(1)
				N1	O2	3x	2.643(9)
					O2	6x	2.925(3)
					N2	3x	3.043(9)

Table S9 Interatomic anion-cation distances of Cr4_HH266, $R\overline{3}m$ after dynamical refinement.

Atom 1	Atom 2	Count	d 1,2 (Å)
Sn1	O2	3x	2.067(6)
	N2	3x	2.175(6)
Ge1	N2	3x	1.862(7)

	N1	1x	1.889(8)
Ge2	O1	2x	1.937(4)
	N2	4x	2.025(7)
Ge3	O2	4x	1.940(5)
	N1	2x	1.995(4)

Table S10 Interatomic cation-cation and anion-anion distances of Cr4_HH266, $R\overline{3}m$ after dynamical refinement.

Atom 1	Atom 2	Count	d 1,2 (Å)	Atom 1	Atom 2	Count	d 1,2 (Å)
Sn1	Ge2	3x	3.140(2)	N2	O1	1x	2.666(9)
	Ge1	3x	3.4919(9)		N2	2x	2.78(1)
	Ge3	6x	3.574(1)		O1	2x	2.933(6)
Ge1	Ge3	3x	3.401(3)		N2	2x	2.948(7)
	Ge2	6x	3.408(2)		O2	2x	3.013(84)
	Sn1	3x	3.4919(9)		N1	1x	3.034(9)
	Ge1	1x	3.505(4)		N2	2x	3.068(7)
Ge2	Ge2	4x	2.9230	O2	N1	1x	2.632(9)
	Sn1	2x	3.140(2)		O2	2x	2.723(8)
	Ge1	4x	3.408(2)		O2	2x	2.763(8)
Ge3	Ge3	4x	2.9230		N1	2x	2.925(3)
	Ge1	2x	3.401(3)		N2	2x	3.013(8)
	Sn1	4x	3.574(1)		O2	2x	3.123(8)
					O2	1x	3.130(9)
				O1	N2	3x	2.666(9)
					N2	6x	2.933(6)
					O2	3x	3.130(9)
				N1	O2	3x	2.632(9)
					O2	6x	2.925(3)

		N2	3x	3.034(9)

Table S11 Acquisition details of the ADT measurements.

	Cr1_HH228	Cr2_HH266	Cr3_HH266	Cr4_HH266
TEM	Tecnai F30 ST	Tecnai F30 ST	Tecnai F30 ST	Tecnai F30 ST
Wavelength (Å)	0.0197	0.0197	0.0197	0.0197
Precession angle (deg.)	1	0.93	0.93	0.93
Tilt step (deg.)	1	1	1	1
No of frames	131	121	121	71
Starting angle (deg.)	-70	-60	-60	-40
Beam size (nm)	200	200	200	200
Camera length (mm)	750	560	1000	560

Table S12 Details of the dynamical refinements

	Cr1_HH228	Cr2_HH266	Cr3_HH266	Cr4_HH266
B(iso) (Å^2)	-0.025	-0.532	-0.154	0.382
g(max) (Å^{-1})	2	1.5	1.6	1.8
smax(matrix)	0.01	0.01	0.01	0.01
smax(refine)	0.1	0.1	0.1	0.1
Limit on Rsg	0.25	0.3	0.35	0.4
D(sg)min	0.0015	0	0	0
Number of integration steps	150	120	120	120
Calculated crystal thickness (Å)	497(3)	584(6)	248(3)	177(4)
Completeness (all)	98.74%	93.9%	98.89%	99.64%
Used reflections all(obs) (obs: I>3σ)	2989(2576)	2369(2275)	1734(1577)	2279(1504)
Parameters	162	140	148	88
Goodness of fit (obs)	3.59	4.91	5.22	4.38
Goodness of fit (all)	3.37	4.83	5.01	3.65

R(obs) / wR(obs)	9.42/10.53	11.25/13.33	11.72/13.79	14.52/16.51
R(all) / wR(all)	10.01/10.64	11.58/13.37	12.43/13.87	15.91/16.24

Table S13 Details of the kinematical refinements

	Cr1_HH228	Cr2_HH266	Cr3_HH266	Cr4_HH266
R_{int} (%)	32.88	23.34	32.31	17.2
B(iso) (Å^2)	-1.08	-1.27	-1.09	-0.65
Completeness (%)	86.6	94.9	90.8	96.6
Used reflections all(obs) (obs: I>3σ)	802(788)	872(465)	355(342)	513(502)
Parameters	31	31	17	17
Goodness of fit (obs)	13.84	5.42	30.57	18.47
goodness of fit (all)	13.72	4.1	29.98	18.27
R(obs) / wR(all)	19.41/21.28	21.28/24.61	30.95/38.14	24.31/30.31
Resolution (Å)	0.5	0.5	0.71	0.625

Table S14 Anisotropic displacement parameters after dynamical refinement of Cr1_HH228, $P6_3mc$

	U11	U22	U33	U12	U13	U23
Sn1	0.0074(5)	0.0074(5)	0.0062(6)	0.0037(3)	0	0
Ge1	0.0057(7)	0.0057(7)	0.0044(8)	0.0028(4)	0	0
Ge2	0.0051(4)	0.0051(4)	0.0066(5)	0.0034(5)	0.0002(2)	-0.0002(2)
N1	0.009(3)	0.009(3)	0.002(4)	0.005(2)	0	0
N2	0.006(2)	0.006(2)	0.002(2)	0.002(2)	-0.0029(9)	0.0029(9)
O1	0.002(2)	0.002(2)	0.004(3)	0.001(1)	0	0
O2	0.011(2)	0.011(2)	0.013(2)	0.009(2)	0.0021(8)	-0.0021(8)

Table S15 Details of the structure solution using SIR2014

	Cr1_HH228	Cr4_HH266

Resolution (Å)	0.8	0.8
Completeness (%)	100	98.02
Ind. reflections	136	248
B(iso) (Å^2)	-1.61	-0.27
R(int) (%)	30.63	20.64

Table S16 Bond valence sums of the Nolanite type phase calculated from Datasets Cr1_HH228 and Cr2_HH266

	Cr1_HH228	Cr2_HH266
Sn1	3.96(2)	3.94(4)
Gesn1		2.62(4)
Ge1	3.86(2)	3.90(5)
Ge2	3.833(16)	4.04(3)
N1	3.10(2)	3.23(5)
N2	3.027(17)	3.12(3)
O1	1.787(10)	1.86(2)
O2	1.783(13)	1.84(2)

The bond valence sum of Gesn1 is lower because its position is mainly occupied by Sn. We suspect that the interatomic distances of this position are locally shorter if a Ge atom occupies this position. Unfortunately, due to the small amount of Ge on that position and the quality of the data, this difference cannot be resolved in the refinement.

Table S17 Bond valence sums of the 6*R* polytype calculated from the datasets Cr3_HH266 and Cr4_HH266

	Cr3_HH266	Cr4_HH266
Sn1	4.28(4)	3.91(3)
Ge1	3.97(4)	4.12(4)
Ge2	3.86(3)	3.90(3)
Ge3	3.77(2)	3.84(2)

N2	3.07(3)	3.06(3)
O2	1.838(18)	1.829(17)
O1	1.798(13)	1.791(11)
N1	3.18(3)	3.18(3)

Table S18 Used bond valence parameters with references

		BV-rho	BV-B	Source
Sn4+	O^{2-}	1.946	0.274	(Gagné & Hawthorne, 2015)
Sn^{4+}	N^{3-}	2.03	0.35	Brown private communication
Ge^{4+}	N^{3-}	1.88	0.37	(Brese & O'Keeffe, 1991)
Ge^{4+}	O^{2-}	1.75	0.363	(Gagné & Hawthorne, 2015)

References

Brese, N. E. & O'Keeffe, M. (1991). Acta Crystallogr B Struct Sci. 47, 192–197, doi:10.1107/S0108768190011041.

Brown, I. D. (2020). Bond valence parameters, https://www.iucr.org/resources/data/datasets/bond-valence-parameters.

Gagné, O. C. & Hawthorne, F. C. (2015). *Acta crystallographica Section B, Structural science, crystal engineering and materials*. **71**, 562–578, doi:10.1107/S2052520615016297.
